# The Urban Liveability Index: developing a policy-relevant urban liveability composite measure and evaluating associations with transport mode choice

**DOI:** 10.1186/s12942-019-0178-8

**Published:** 2019-06-11

**Authors:** Carl Higgs, Hannah Badland, Koen Simons, Luke D. Knibbs, Billie Giles-Corti

**Affiliations:** 1 NHMRC Centre of Research Excellence in Healthy Liveable Communities, Centre for Urban Research, RMIT University, Room 12, Building 15, Level 3 RMIT University, 124 Latrobe St, Melbourne, VIC Australia; 2 Centre for Urban Research, RMIT University, Melbourne, VIC Australia; 3 Centre for Epidemiology and Biostatistics, University of Melbourne, Melbourne, VIC Australia; 4 School of Public Health, University of Queensland, Brisbane, QLD Australia

## Abstract

**Background:**

Designing healthy, liveable cities is a global priority. Current liveability indices are aggregated at the city-level, do not reflect spatial variation within cities, and are often not aligned to policy or health.

**Objectives:**

To combine policy-relevant liveability indicators associated with health into a spatial Urban Liveability Index (ULI) and examine its association with adult travel behaviours.

**Methods:**

We developed methods to calculate spatial liveability indicators and the ULI for all residential addresses in Melbourne, Australia. Associations between the address-level ULI and adult travel behaviours from the 2012–2014 Victorian Integrated Survey of Travel and Activity (VISTA) (*n* = 12,323) were analysed using multilevel logistic regression. Sensitivity analyses to evaluate impact of methodological choices on distribution of liveability as assessed by the ULI and associations with travel mode choice were also conducted.

**Results:**

Liveability estimates were calculated for 1,550,641 residential addresses. ULI scores were positively associated with active transport behaviour: for each unit increase in the ULI score the estimated adjusted odds ratio (OR) for: walking increased by 12% (95% Credible Interval: 9%, 15%); cycling increased by 10% (4%, 17%); public transport increased by 15% (11%, 19%); and private vehicle transport decreased by 12% (− 9%, − 15%).

**Conclusions:**

The ULI provides an evidence-informed and policy-relevant measure of urban liveability, that is significantly and approximately linearly associated with adult travel behaviours in the Melbourne context. The ULI can be used to evaluate progress towards implementing policies designed to achieve more liveable cities, identify spatial inequities, and examine relationships with health and wellbeing.

**Electronic supplementary material:**

The online version of this article (10.1186/s12942-019-0178-8) contains supplementary material, which is available to authorized users.

## Introduction

City planning originated as a means of protecting the public’s health in crowded and polluted industrialising cities; however, the links between planning and health have attenuated over the last century [1]. More recently, creating ‘liveable cities’ is a growing policy aspiration across multiple levels of government globally [2]. This is largely in response to population projections, rapid urbanisation, and climate change, whereby designing liveable cities that promote health and wellbeing is now a global priority, as realised, for example, through the UN Sustainable Development Goals [3]. In Australia, examples include the Federal Government’s National Cities Performance Framework [4], the state of Victoria’s Plan Melbourne [5], and Cardinia Shire’s Liveability Plan [6].

Despite increasing use of the concept of ‘liveability’ and its intuitive meaning, it is rarely explicitly defined. Following a literature review, we developed a comprehensive definition of urban liveability [7] as being communities that are “safe, attractive, socially cohesive and inclusive, and environmentally sustainable; with affordable and diverse housing linked by convenient public transport, walking and cycling infrastructure to employment, education, public open space, local shops, health and community services, and leisure and cultural opportunities”. This definition reflects the social determinants of health and wellbeing [8] and implies the need for integrated urban governance across multiple sectors to create a healthy liveable city [9]. To evaluate and inform urban policy, we identified a need for policy-relevant liveability indicators that are: aligned with urban planning policies; measured at appropriate geographic scales to minimise ecological bias; and, linked with population health datasets to examine associations with health and wellbeing outcomes [7, 10]. In this way, the concept of urban liveability could be used to inform and evaluate urban planning policies that create health promoting cities [9].

To create appropriate liveability indicators, we embarked on a research program that conceptualized and tested hypothesized pathways through which multiple domains of liveability influence health and wellbeing outcomes. Where possible these were aligned with urban planning policy, and when no policies existed, the indicators were developed based on empirical evidence. Details of this foundational work are described elsewhere [10–21]. Our findings were generally consistent with a growing body of international evidence observing positive associations between various liveability domains and the health and wellbeing of adults [22, 23].

Building on this program of research, in this paper we consider the combined influence of these underlying domains of urban liveability—transport, social infrastructure, employment, walkability, housing and green infrastructure—to assess how ‘liveable’ cities might enhance health and wellbeing and reduce spatial inequities. Indeed, creating a robust composite tool could help identify spatial variability of urban liveability across cities and better inform decision-makers of the overall impact of integrated city planning policies. It achieves this by (1) summarising and visualising aspects of the build environment based on a transparent and reproducible method and (2) enabling research into the association between the indicator and health outcomes of interest.

This paper presents the development of the Urban Liveability Index (ULI), an evidence-informed, policy-relevant liveability index that incorporates indicators of liveability found previously to support health and wellbeing. It has been designed to capture the spatial distribution of within-city variation so that any inequities in urban liveability may be assessed quantitatively and visually.

Our emphasis is on within-city variation in liveability to identify inequities in the provision of urban infrastructure, and hence requires a high resolution approach. To illustrate this, we compare resolution-specific estimates of association between ULI and travel behaviour in adults. We further use this case study to examine the influence of other methodological choices through a series of sensitivity analyses.

## Methods

### Calculating liveability for residential lots

Construction of the ULI was informed by a guide for composite indicator construction published by the Organisation for Economic Co-operation and Development (OECD) [24], which proposed an iterative process: theoretical framework development; data collation, cleaning and analysis; normalisation; weighting and aggregation; evaluation of robustness and sensitivity; post-evaluation revision of processes in earlier steps as required; analysis with other variables; and visualisation and dissemination. In the theoretical framework stage, we drew upon our previous conceptual work [25] to map the relationship between the underlying liveability domains, data sources which were accessible in order to measure these in the form of derived indicators, and how these indicators may be combined to form a composite index.

**Fig. 1:**
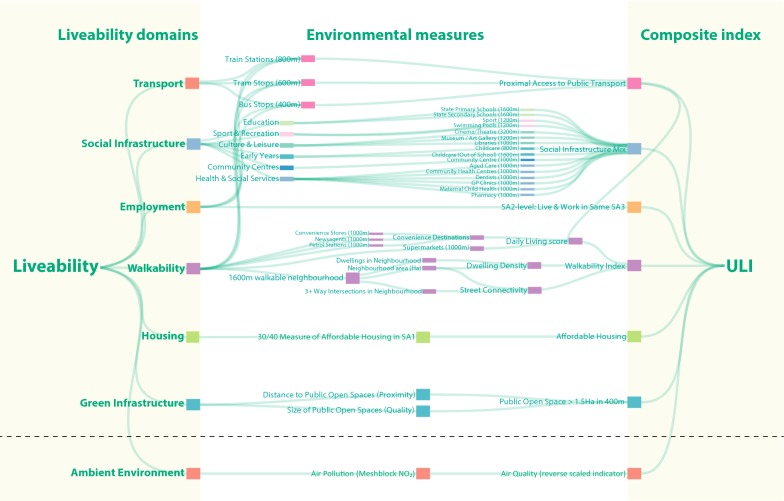
Liveability domains and indicators as conceptualised in the Urban Liveability Index. A conceptual flow diagram of our process, from the concept of liveability (left) considered through key domains, neighbourhood measures which are ultimately combined in the ULI (right). The inclusion of an air quality indicator (below dashed line) in the liveability model was evaluated as a sensitivity analysis

The final set of spatial liveability indicators chosen for inclusion in the ULI reflected a balance of parsimony (reflecting the core aspects of liveability identified through our previous work) and pragmatism (i.e., what data could be readily accessed for Melbourne, but also for other urban and regional locations of interest across Australia). We acknowledge these indicators do not capture the full complexity of liveability; however, they broadly reflect the social determinants of health, which are important for health and wellbeing [23]. Our conceptual model of indicators as grouped by liveability domain is presented in Fig. 1 and methods of calculation are described below.

The spatial liveability indicators and the ULI were calculated using processes scripted using Python 2.7.14 [38] interfacing with ArcPy/ArcGIS 10.5 software with the Network Analyst extension [39] and a PostgreSQL 9.6 database with the PostGIS 2.3.1 extension [40, 41].

#### Study region

The ULI was calculated for Melbourne, the capital of the Australian state of Victoria. Melbourne’s population in 2016 was 4.7 million people [36], representing almost 20% of the Australian population, and is expected to grow to around 8 million by 2050 [37]. We restricted the study area to the metropolitan region corresponding to the Australian Bureau of Statistics (ABS) statistical division for Melbourne in 2011 [32], where this intersected the ABS Sections of State geographic classification of ‘Urban’ or ‘Other Urban’ [73]. The time point of 2011 aligned with the completion of the five-yearly national census in 2011, and for which other appropriate spatial data were also available. To mitigate edge effects, we used a 10 km study region buffer for expanded destination and road network coverage. This allowed for residential addresses on the study region periphery to have adequate network connectivity to proximal destinations that may not be in the study region itself.

#### Sampling locations

Measures of local neighbourhood liveability were calculated for a set of 1,550,641 sample point locations across Melbourne. These sampling points were derived from the Geocoded National Address File (G-NAF) for 2012 [42], a regularly updated database of all addresses within Australia. Address points in the study region (n = 2,259,075) were dissolved by location, such that if a series of addresses shared the same coordinates these were represented as a single point. We refer to these sampling points as residential lots since they serve as proxy locations for lots with residential potential.

To ensure G-NAF address points were representative of where people actually live, address points were only retained if they were located within a Mesh Block [72] with 2011 dwelling count [74] greater than zero; otherwise, they were excluded. Mesh Blocks are the smallest geographical unit used by the ABS [43], with a median dwelling count within our metro-urban Melbourne study region of 19 (interquartile range, IQR 0–37). Address points located in Statistical Area 1 regions (SA1s; comprising approximately 200 to 800 persons, akin to a local neighbourhood [72]) for which the ABS 2011 Socio-economic Indexes for Areas (SEIFA) Index of Relative Socio-economic Disadvantage (IRSD) [75] was not calculated were excluded. This served as an implicit adoption of the IRSD exclusion criteria [43], which is not calculated for SA1s with: a population of 10 persons or fewer; five or fewer employed persons; five or fewer classifiable occupied private dwellings; 20% or lower occupancy of private dwellings; no addresses; or located offshore.

In addition to the geographical and topological exclusions, any residential lot with a null record following calculation were also excluded. Null records for a local built environment indicator could occur for residential lots in locations without proximal access to a walkable road network. In total, 48 residential lots across 8 Mesh Blocks were excluded due to inability to calculate at least one local neighbourhood indicator (destination access, or walkability index); 3936 residential lots across 117 Mesh Blocks were excluded due to being located in an SA1 for which the SEIFA IRSD had not been calculated by the ABS.

The final set of 1,550,641 residential lots with valid indicators served as sampling locations for the spatial distribution of liveability indicators and in turn the ULI; when aggregated these corresponded to 42,154 Mesh Blocks, 8958 SA1 regions and 31 Local Government Areas (LGAs)across metropolitan Melbourne. The counts at successive stages of processing are enumerated in Table 1.

**Table 1:** Residential lot counts at successive stages of processing, from input to final ULI estimates

Geographic scale	Processing stage	*n*
Disaggregated points		
*Residential lot*	2012 G-NAF (Victoria)	3,238,149
	Within metropolitan Melbourne	2,259,075
	Within Mesh Blocks with 2011 dwelling count > 0	2,095,669
	Duplicate coordinates collapsed to unique location	1,554,624
	In SA1 with IRSD	1,550,688
	With valid indicators	1,550,641
Aggregate areas		
*Mesh Block*	2011 ABS data (Victoria)	81,377
	Within metropolitan Melbourne	52,128
	With > 0 dwellings	44,581
	Associated with addresses with valid indicators	42,154
*Statistical Area 1 (SA1)*	2011 ABS data (Victoria)	13,339
	Within metropolitan Melbourne	9404
	With IRSD	9115
	Associated with addresses with valid indicators	8958
*Local Government Areas (LGA)*		
	2011 ABS data (Victoria)	81
	Within metropolitan Melbourne	33
	Associated with addresses with valid indicators	31

Counts for the administrative boundaries residential lots correspond to when aggregated are also presented

#### Calculating spatial indicators of liveability

A cleaned, ‘walkable’ road network that excluded highways and freeways was created [44] based on the 2012 Vicmap Transport dataset [76]. The local walkable neighbourhood for each residential lot was evaluated as its 1600 m network extent, with network lines buffered by 50 m width [45]. Liveability indicators were calculated as follows, with further detail on methods used for evaluating destination access provided further below. The liveability indicators for residential lots were measured at the finest scale possible given available data and destination data were compiled from various sources dated as close as possible to 2011.

*Walkability* was calculated as the sum of standardised scores of local neighbourhood street connectivity, dwelling density and daily living score [14, 26]. Street connectivity was calculated as the ratio of intersections [76] to local walkable neighbourhood area in square kilometres. Dwelling density was calculated as the total number of dwellings located in Mesh Blocks intersecting each participants’ local walkable neighbourhood divided by the neighbourhood size in hectares. A daily living score ranging from 0 to 3 for access to three kinds of basic amenities (a public transport stop, a supermarket, and a convenience location) was calculated drawing on the method of Badland et al. [14].

*Social infrastructure mix* was calculated as a score for local access to 15 different types of destination across domains of ‘early years’, ‘education’, ‘community, culture and leisure’, ‘health and social services’ and ‘sport and recreation’ following the method of Davern et al. [17].

*Public transport access* was evaluated based on Victorian government planning standards for public transport access within mode-specific threshold distances of either a: bus stop within 400 m, tram stop within 600 m or train station within 800 m [27].

*Large public open space (POS) access* was based on a Victorian policy standard that POS should be available within 400 m of a residence [27]. Our measure included both proximity and size (larger than 1.5 ha), based on previous research indicating these dimensions’ importance for supporting recreational walking [28–31].To capture the accessibility of POS—which often can be entered from any point—polygon boundaries [77] were converted to point vertices at 50 m intervals [29].

*Affordable housing* was based on the proportion of low income households (in the bottom 40 per cent of the Australian income distribution) not experiencing housing affordability stress. Housing affordability stress was defined as the proportion of low income households paying more than 30 per cent of their income on housing costs [13]. This indicator was calculated at the SA1 level, based on a 2011 Census custom data report provided by the ABS [78].

*Local work opportunities* was calculated as the proportion of employed persons who live and work in the same Statistical Area 3 (SA3; a regional division of approximately 30,000 to 130,000 persons [32]), calculated at the Statistical Area 2 level (SA2; an average of 10,000 persons [32], akin to a socially and economically coherent local community).

In our previously published conceptual model of the influence of urban and transport design and planning on health and wellbeing [7, 9], air quality was considered an outcome of city planning decisions, whereby integrated planning influences transport mode and emissions. However, given global concerns about the adverse health effects of air pollution exposure in cities [33, 34] and following feedback from our international project advisory group, it was decided to trial inclusion of an air quality indicator in the ULI.

Hence, a sensitivity analysis was conducted to examine the impact including an *air quality* indicator in the ULI. The 2011 Melbourne segment of a national, longitudinal ambient air pollution dataset derived from a satellite-based land-use regression model was acquired featuring Mesh Block-level annual average prediction of nitrogen dioxide (NO_2_) [79]; the surface model was validated to predict 69% of spatial variation in annual NO_2_ (root mean square error 25%) [80]. Mesh Block-level modelled annual average NO_2_ serves as a general proxy for traffic-related and other combustion-derived air pollutants. Australian air quality standards prescribe a threshold of 30 parts per billion (ppb) for annual average NO_2_ concentrations [35]. As higher levels of air pollution are less desirable, the reverse-scaled air pollution measure was used as an indicator of air quality, to match the polarity of the other indicators.

#### Evaluating destination access

Distance to destinations along a street network was measured using origin–destination (OD) matrices processed using ArcGIS Network Analyst [38]. Destination accessibility was considered with reference to destination specific threshold distances within which access should be achieved [17], summarised in Table 2.

**Table 2:** ULI indicator destinations with access cut-off distances and access summary for Melbourne residential lots

Measure	Destination	Cut-off distance (m)	Median [IQR] distance (m)
*Daily living (/3)*	Public transport stop [76] (at least one)		
	Bus stop	400	318 [180,513]
	Tram stop	600	8342 [2294,19833]
	Train station	800	2217 [1232,3973]
	Food (supermarket [81])	1000	1181 [759,1715]
	Convenience [81] (at least one)		
	Convenience store	1000	812 [485,1319]
	Petrol station	1000	1098 [689,1702]
	Newsagent	1000	1330 [794,2251]
*Social infrastructure mix (/15)*	Community, culture and leisure [82]		
	Community centre	1000	2082 [1242,3268]
	Library	1000	2232 [1387,3524]
	Museum/art gallery	3200	4649 [2645,7651]
	Cinema/theatre	3200	3471 [2064,7544]
	Early years [83]		
	Childcare	800	659 [420,971]
	Childcare (outside school hours)	1600	984 [647,1449]
	Education [82]		
	State secondary school	1600	1857 [1219,2709]
	State primary school	1600	1036 [698,1470]
	Health and social services [82]		
	Aged care	1000	1065 [623,1795]
	Community health centre	1000	1715 [1037,2843]
	Dentist	1000	915 [526,1553]
	GP clinic	1000	901 [546,1412]
	Maternal/child health centre	1000	2045 [1242,3368]
	Pharmacy	1000	892 [548,1414]
	Sport [82]		
	Sport	1200	868 [572,1289]
	Swimming pool	1200	2963 [1919,4322]

Thresholds readily lead to the creation of binary (‘no’ or ‘yes’) indicators of destination access; an intuitive way to express whether a policy has or has not been achieved, which when aggregated is interpreted as the proportion of residential lots within that area that have access within the policy-recommended distance. However, the imposition of a hard threshold (for example, access to a bus stop within 400 m) is an arbitrary choice that results in loss of information that can otherwise be used for statistical inference [46]. When a hard threshold is applied, living within 390 m of a bus stop gives a person full benefit, yet a resident living at 410 m receives zero benefit, despite the modest difference in distance. Further, while acknowledging that reality may be more complex, our model assumes that greater proximity is preferable.

**Fig. 2:**
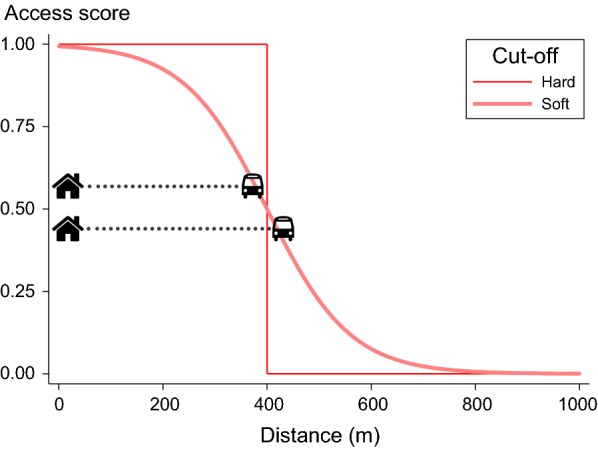
Illustrative comparison of the hard and soft thresholds for access to a bus stop within 400 m of a residential address

An approach that recognises the value of greater proximity was considered important for capturing the nuances of liveability. We developed a ‘soft threshold’ approach to evaluating destination access using a logistic decay function, which yields a continuous access score for each destination type ranging from 0 to 1, as an alternative to binary hard thresholds:

$${\text{Soft threshold access score}} = \frac{1}{{1 + exp\left( {k\frac{{d_{q} - c_{q} }}{{c_{q} }}} \right)}}$$

where ${d_{q}}$ is the pedestrian network distance to nearest destination $q$, $c_{q}$ is the destination-specific threshold distance and $k$ adjusts the slope of decay, which was set as $k = 5$. Figure 2 illustrates calculation of access scores for a bus stop using hard and soft thresholds, with a policy-relevant threshold of 400 m. An illustrative overlay of two scenarios at 390 m and 410 m shows how the access scores for distance to closest bus stop from a residential lot are similar when using the soft threshold (respectively, 0.53 and 0.47), but at polar extremes when using the hard threshold (0 and 1).

The area level averages of the hard- and soft-threshold estimates would be expected to be more or less similar, however they differ with regard to interpretation: the hard-threshold average of an area is the proportion of lots with access to a destination within the threshold distance; the soft-threshold average may be more appropriately considered a score of ‘effective access’ to a destination for that area, given the recommended threshold.

The liveability indicators used in the ULI were calculated using soft-thresholds for destination access where applicable. Alternate versions of the liveability indicators and the ULI were calculated using hard thresholds and evaluated as a sensitivity analysis.

#### Urban Liveability Index creation

The formulae presented below conceptualise the pre-calculated indicator data used to construct the ULI as a matrix $\varvec{X}$ with $n$ rows of unique residential address points indexed as $i$, and 7 columns of indicators indexed as $j$. The value of indicator $j$ at address $i$ is $x_{ij}$, while the set of values across all addresses for indicator $j$ is $\varvec{x}_{j}$ (in bold). The mean and population standard deviation for indicator $j$ are written respectively as $\bar{x}_{j}$ and ${\text{s}}_{{\varvec{x}_{j} }}$ (as defined below). The average of all indicators at address $i$ is referred to as $\bar{x}_{i}$. Subsequent formulae will expand upon these concepts.

**Fig. 3:**
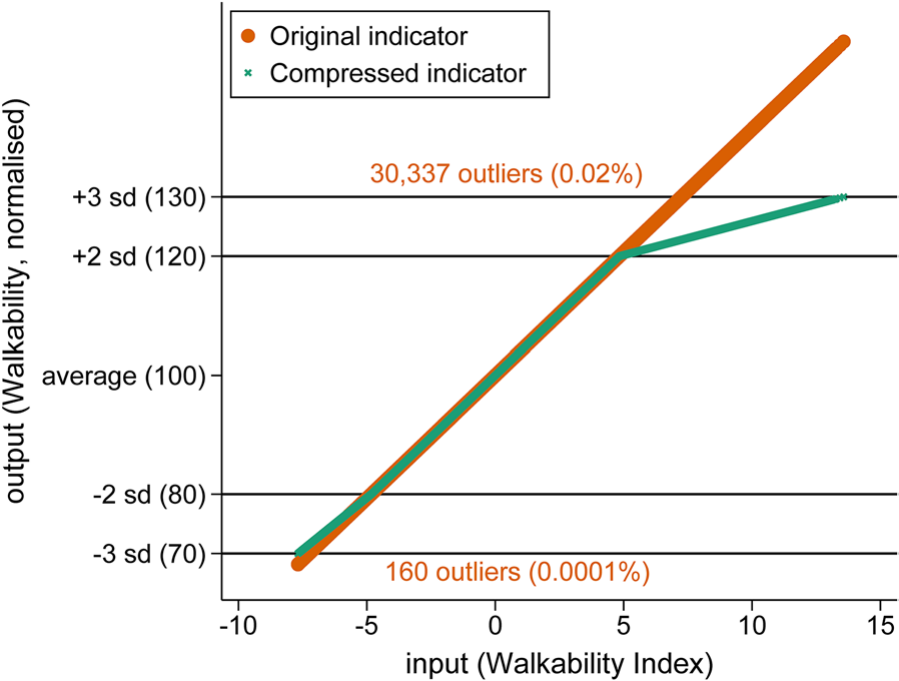
Example of compression algorithm for scaling outliers to within 3 standard deviations of the indicator mean

The ULI was constructed based on the Mazziota–Pareto Index (MPI) method, an approach designed for use in contexts of either development or deprivation [48]. In these contexts, as with that of urban liveability, indicators relate to discrete domains and are considered non-compensable: that is, scoring high on one or two within a suite of indicators should not wholly compensate for poor performance on the rest (and vice versa). The MPI method involves standardisation of indicators such that they have a usual range of $\pm 3$ standard deviations around a mean; variation across the set of indicators to be combined is penalised, providing a tacit incentive for balanced performance. Indicators were conditionally scaled prior to ULI construction to constrain the influence of extreme outliers. This was done to limit the impact of an excessive penalty arising from an isolated extreme indicator result in an otherwise balanced and well performing set; we considered it appropriate for some penalty to be applied, but since the penalty is non-linear it was considered desirable to enforce scaling to the stable usual range described in the examples of Mazziota and Pareto [48]. We developed and applied an algorithm which scaled address values for any indicator $\varvec{x}_{j}$ which had a value $x_{ij}$ more than three standard deviations $s_{{\varvec{x}_{j} }}$ from the indicator mean $\bar{x}_{j}$. to lie within this range by applying a linear transformation in such cases only to those values more than two standard deviations distance from the mean (Fig. 3):

$$\bar{x}_{j} = \frac{1}{N}\mathop \sum \limits_{i = 1}^{N} x_{ij}$$

$${\text{s}}_{{\varvec{x}_{j} }} = \sqrt {\frac{1}{N}\mathop \sum \limits_{i = 1}^{n} \left( {x_{ij} - \bar{x}_{j} } \right)^{2} }$$

$$L_{j} = \bar{x}_{j} - 2s_{{\varvec{x}_{j} }}$$

$$U_{j} = \bar{x}_{j} + 2s_{{\varvec{x}_{j} }}$$

$$x_{ij}^{\prime } = \left\{ {\begin{array}{*{20}l} L_{j} + s_{{x_{j} }} \frac{{x_{ij} - {\rm min} \left( {x_{j} } \right)}}{{L_{j} - {\rm min} \left( {x_{j} } \right)}}, &\quad {\text{if}}\,({\rm min} \left( {x_{j} } \right) < L_{j} - s_{{x_{j} }} ) \cup (x_{ij} < L_{j} ) \\ U_{j} + s_{{x_{j} }} \frac{{x_{ij} - U_{j} }}{{{\rm max} \left( {x_{j} } \right) - U_{j} }}, &\quad {\text{if}}\,({\rm max} \left( {x_{j} } \right) > U_{j} + s_{{x_{j} }} ) \cup (x_{ij} > U_{j} ) \\ x_{ij} , &\quad {\rm otherwise} \\ \end{array} } \right.$$

This conditionally applied transformation is analogous to signal processing used to boost low amplitude signals and compress those that are too ‘loud’ towards a narrower range [47] facilitating balance of the overall ensemble of indicators in the ULI.

The conditionally scaled residential address estimates for each indicator ($x_{ij}^{'}$) were normalised as part of the MPI construction process using the following formula [48]:

$$z_{ij} = \left\{ {\begin{array}{*{20}c} {\begin{array}{*{20}l} {100 + 10\frac{{x_{ij}^{\prime } - \bar{x}_{j}^{\prime } }}{{s_{{x_{j}^{\prime } }} }}, \quad {\text{if polarity is positive}} } \\ \end{array} } \\ {100 - 10\frac{{x_{ij}^{{\prime }} - \bar{x}_{j}^{\prime } }}{{s_{{x_{j}^{'} }} }} , \quad {\text{if polarity is negative }}} \\ \end{array} } \right.$$

Each $z_{ij}$ represents the conditionally scaled and normalised score $z$ for residential address $i$ with regard to the indicator $j$. This results in column-wise per-indicator means of $100$ and standard deviation $10$. An expected usual range of from 70 to 130 was enforced through our approach to outlier management.

The composite indicator for each residential address (${\text{ULI}}_{i} )$ was calculated as the row-wise average of all indicators for that address $\bar{z}_{i}$ minus a penalty, which rewarded a balanced set of indicators. This penalty is defined as the variability of sub-indicators at the residential address $\left( {s_{{\varvec{z}_{i} }}^{2} } \right)$ relative to the average of all indicators at the specific address $\left( {\bar{z}_{i} } \right)$.

$$\bar{z}_{i} = \frac{1}{{N_{j} }}\mathop \sum \limits_{j = 1}^{N} z_{ij}$$

$$s_{{\varvec{z}_{i} }} = \sqrt {\frac{{\mathop \sum \nolimits_{j = 1}^{n} \left( {z_{ij} - \bar{z}_{i} } \right)^{2} }}{{N_{j} }}}$$

$${\text{ULI}}_{i} = \bar{z}_{i} - \frac{{s_{{\varvec{z}_{i} }}^{2} }}{{\bar{z}_{i} }}$$

### Testing associations between the ULI and travel mode choice

We investigated the association between the ULI and travel mode choice for trips within a person’s residential neighbourhood using the Victorian Integrated Survey of Travel Activity 2012–14 (VISTA12). VISTA12 provides a geographically representative survey of travel behaviour for the Victorian population. Survey data were obtained from the Victorian Department of Transport, Planning and Local Infrastructure (project ethics approval number CHEAN A 20582-03/17). Participants in VISTA12 recorded all trips, stops, and the modes used on one survey day, and provided socio-demographic information. VISTA12’s sampling consisted of randomly selected Mesh Blocks and multiple households sampled within each Mesh Block. All household members were asked to participate. The VISTA12 data set contained 22,934 unique people within Melbourne who recorded 73,889 trips. Each VISTA12 geocoded household address point was associated with a ULI score based on linkage with the closest residential address.

#### Defining eligible trips

Participants aged 18 years and older who travelled on the survey day, had eligible trips starting or ending within their residential neighbourhood (< 1600 m of home address), and who had complete data for all covariates were included in the final sample. Eligible trips were defined as non-recreational trips (i.e. those that did not both start and end in ‘‘parks’’, ‘‘forests’ or ‘lakes’). Following the approach of Badland et al. [14], for all eligible trips the primary mode of travel was categorised as ‘walking’, ‘cycling’, ‘driving’ or ‘public transport’, and, for each participant four binary indicators were derived, representing at least one eligible trip of each travel mode.

#### Covariates

Socio-demographic variables included age group, sex, household income, household type, vehicle ownership, and employment status. Household income was transformed into equivalised income using the modified-OECD scale, adjusting for the number of adults and children living in the household [49]. Univariable associations between covariates and ULI quintiles were evaluated using linear models with application of a square root transformation in case of skewed distribution, or Chi squared tests for categorical variables.

#### Statistical modelling

VISTA12’s nested sampling frame of people within households, within Mesh Blocks necessitated a multilevel model. Separate multilevel logistic models were fitted for each of the outcome variables: walking, cycling, taking public transport and driving at least once on the day of the survey. All models were adjusted for socio-demographic variables, and an indicator variable was used for the day of the week. Age was grouped into 18–29, 30–49, 50–64, and ≥ 65 years. Statistical analyses were undertaken using R version 3.5.1 [50], and package ‘rstanarm’ version 2.17.4 [51]. The formula used for all analyses was of the form:


formula <- binary_outcome ~ measure_of_interest + agegroup + sex + factor(day_of_week) + has_any_work + household_type + vehicle + oecd_income_quintile + (1|meshblock/household)


where binary outcome refers to one of four separately analysed travel modes, and measure of interest relates to the ULI or its separately analysed constituent indicators. Covariates were included as fixed effects, with household nested within Mesh Blocks as random effects.

Multilevel logistic regression models using this formula were implemented with default, weakly informative priors using the following code:

model < - stan_glmer(formula, family = binomial, data = db, chains = 6)

### Sensitivity analysis

Sensitivity analyses were conducted to evaluate the impact on spatial distribution of liveability and any change in estimates of association with travel mode choice related to methodological aspects, being the: (1) inclusion of an air quality indicator (reverse-scaled NO_2_ air pollution) in the ULI; and (2) use of aggregate areas instead of residential lots.

The ULI was calculated for individual locations rather than areas in the first instance, allowing for evaluation of the spatial distribution of liveability and consideration of questions of equity. To evaluate the impact of aggregation, the ULI and its constituent indicators (including air quality) were aggregated to Mesh Block, SA1, Suburb and Local Government Area (LGA) levels using residential lot averages.

We also examined the change in distribution of liveability indicators and the ULI when using hard—instead of soft thresholds for destination access.

## Results

### Liveability distribution for residential lots

**Fig. 4:**
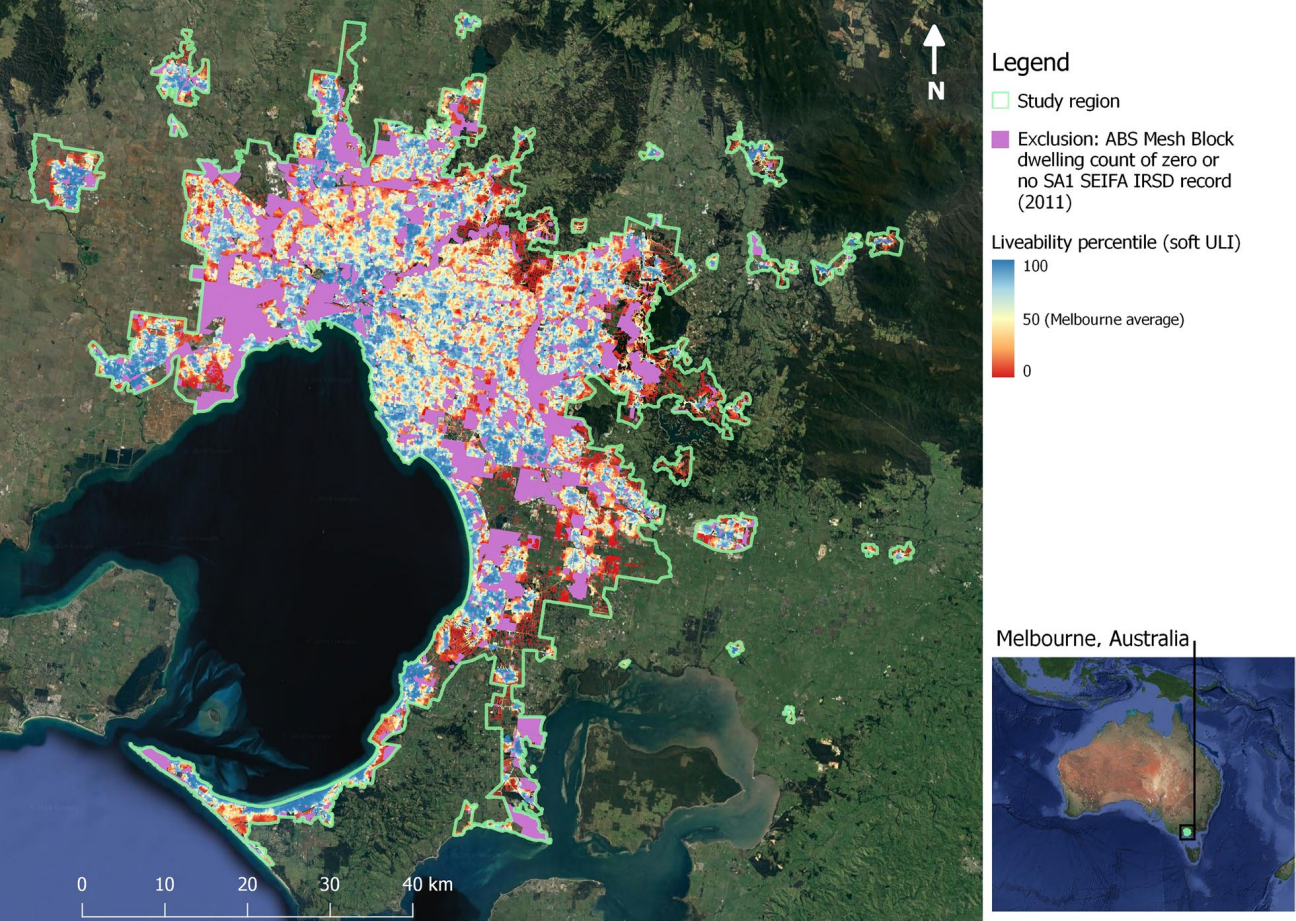
Spatial distribution of the ULI. The spatial distribution of percentiles of ULI for residential lots across Melbourne. The study region (main map), was restricted to the urban portion of the Melbourne statistical division in the state of Victoria, Australia (inset)

Table 2 summarises the median and interquartile range of distance in metres for residential lot access to destinations. Distribution summaries for the residential lot estimates of spatial liveability indicators are presented in Table 3 for the three key stages of data processing leading to ULI construction: (1) raw data; (2) following conditional scaling of outliers to no more than three standard deviations of the indicator mean; and (3) following normalisation to a common scale. The spatial distribution of the ULI in Melbourne for 2011 is presented in Fig. 4; Additional file 1 provides an animated sequence allowing for comparison of choropleth maps of the spatial distribution of quartiles of the ULI with- and without air quality included, and the raw forms of each of its constituent indicators, with aggregation at the SA1 small area scale.

**Table 3:** Summary of liveability indicator and ULI (in italics) distribution for residential lots

	Indicatorestimates					
	Mean ± SD	25th p.	Median	75thp.	Min.	Max.
*Raw indicators*						
Walkability	0.0 ± 2.4	− 1.3	− 0.1	0.9	− 7.7	13.6
Daily Living (/3)	1.7 ± 0.8	1.1	1.8	2.4	0.0	3.0
Dwellings per Ha	14.1 ± 5.8	11.1	13.3	15.7	0.0	52.1
3 + way street connections per km^2^	70.2 ± 20.5	60.3	67.4	76.2	2.0	201.2
Social infrastructure mix (/16)	6.8 ± 3.1	4.4	6.9	9.1	0.0	15.3
Public transport access meets policy (%)	64.5 ± 36.1	32.2	81.9	95.5	0.0	99.3
Large park access (%)	48.2 ± 39.9	4.1	47.2	91.9	0.0	99.3
Air pollution	8.9 ± 2.6	7.1	8.7	10.3	3.6	34.9
Affordable housing (SA1 %)	11.4 ± 8.9	5.4	10.5	16.2	0.0	100.0
Local work opportunities (SA2 %)	26.6 ± 11.2	19.4	24.4	29.7	10.0	68.7
*Conditionally scaled indicators*						
Walkability	− 0.1 ± 2.1	− 1.3	− 0.1	0.9	− 7.2	7.3
Daily Living (/3)	1.7 ± 0.8	1.1	1.8	2.4	0.0	3.0
Dwellings per Ha	13.9 ± 4.9	11.1	13.3	15.7	0.0	31.4
3 + way street connections per km^2^	69.2 ± 17.0	60.3	67.4	76.2	8.7	131.6
Social infrastructure mix (/16)	6.8 ± 3.1	4.4	6.9	9.1	0.0	15.3
Public transport access meets policy (%)	64.5 ± 36.1	32.2	81.9	95.5	0.0	99.3
Large park access (%)	48.2 ± 39.9	4.1	47.2	91.9	0.0	99.3
Air pollution	8.8 ± 2.3	7.1	8.7	10.3	3.6	16.6
Affordable housing (SA1 %)	11.1 ± 8.2	5.4	10.5	16.2	0.0	38.2
Local work opportunities (SA2 %)	26.2 ± 10.2	19.4	24.4	29.7	10.0	60.1
*Normalised, conditionally scaled indicators, with composite index*						
ULI	*99.2. ± .5.0*	*96.1*	*99.5*	*102.6*	*82.1*	*119.7*
ULI including air quality*	*99.2 ± 3.9*	*96.7*	*99.3*	*101.7*	*83.8*	*113.5*
Walkability	100.0 ± 10.0	94.3	99.7	104.7	66.5	134.3
Daily living (/3)	100.0 ± 10.0	92.5	101.5	108.5	79.7	115.0
Dwellings per Ha	100.0 ± 10.0	94.4	98.9	103.8	71.7	135.9
3 + way street connections per km^2^	100.0 ± 10.0	94.8	99.0	104.1	64.3	136.8
Social infrastructure mix (/16)	100.0 ± 10.0	92.5	100.5	107.4	78.4	127.2
Public transport access meets policy (%)	100.0 ± 10.0	91.1	104.8	108.6	82.1	109.7
Large park access (%)	100.0 ± 10.0	89.0	99.7	110.9	87.9	112.8
Air quality	100.0 ± 10.0	93.5	100.5	107.4	66.2	122.7
Affordable housing (SA1 %)	100.0 ± 10.0	93.0	99.3	106.3	86.4	133.2
Local work opportunities (SA2 %)	100.0 ± 10.0	93.3	98.2	103.4	84.1	133.2

Constituent sub-indicators contributing to walkability score are included for reference purposes

* Sensitivity analysis

### Associations between the ULI and travel mode choice

After excluding VISTA12 participants outside of the study area and trips that did not either start or end in their residential neighbourhood, 50,128 eligible trips were retained. Of these, 111 had missing data on mode choice; no trips were identified as recreational. Using the remaining 50,017 eligible trips, we identified 15,917 unique persons with at least one eligible trip, of whom 12,557 were aged ≥ 18 years. A total of 12,323 adult participants with complete data for all covariates were included in the final sample. The average age of included participants was 46.6 ± 16.4 years. There was a balanced sex distribution (6318 females, 51%), and 71% of respondents reported being employed (*n* = 8748). The distribution of included participant covariates is summarised by ULI quintile in Table 4.

**Table 4:** Summaries of covariates and outcomes of retained VISTA12 participants by liveability score quintile

	ULI quintiles					*p* value
	84.6, 95.0	95.0, 98.4	98.4, 100.8	100.8, 103.4	103.4, 117.7	
*N*	2465	2464	2465	2464	2465	
*Age*						
Continuous—median [IQR]	46 [24.0]	48 [25.0]	46 [25.0]	46 [26.0]	43 [25.0]	0.029^a^
Group—n (%)						
18–29	434 (17.6)	407 (16.5)	429 (17.4)	436 (17.7)	469 (19.0)	< 0.001^b^
30–49	981 (39.8)	916 (37.2)	1012 (41.1)	966 (39.2)	1012 (41.1)	
50–64	721 (29.2)	727 (29.5)	649 (26.3)	627 (25.4)	641 (26.0)	
65 and over	329 (13.3)	414 (16.8)	375 (15.2)	435 (17.7)	343 (13.9)	
*Sex*						
Male	1211 (49.1)	1207 (49.0)	1202 (48.8)	1192 (48.4)	1193 (48.4)	0.516^c^
Female	1254 (50.9)	1257 (51.0)	1263 (51.2)	1272 (51.6)	1272 (51.6)	
Household structure						
Lone person	143 (5.8)	239 (9.7)	259 (10.5)	306 (12.4)	436 (17.7)	< 0.001^b^
With children	824 (33.4)	785 (31.9)	753 (30.5)	631 (25.6)	605 (24.5)	
Without children	1498 (60.8)	1440 (58.4)	1453 (58.9)	1527 (62.0)	1424 (57.8)	
*Employed*—*n (%)*						
Yes	1825 (74.0)	1745 (70.8)	1729 (70.1)	1712 (69.5)	1737 (70.5)	0.003^c^
*Vehicle access*—*n (%)*						
No	24 (1.0)	42 (1.7)	80 (3.2)	90 (3.7)	181 (7.3)	< 0.001^c^
*Survey administration day of week*^*e*^—*n (%)*						
Weekday	1922 (78.0)	1868 (75.8)	1834 (74.4)	1883 (76.4)	1878 (76.2)	0.003^b^
Weekend	543 (22.0)	596 (24.2)	631 (25.6)	581 (23.6)	587 (23.8)	
*Equivalised household income (pre*-*tax weekly $AUD)*						
Continuous—median [IQR]	972.2 [775.0]	945 [847.9]	933.3 [833.3]	958.3 [916.7]	1022 [1104.0]	0.098^d^
Quintiles—n (%)						
< 500	448 (18.2)	525 (21.3)	548 (22.2)	552 (22.4)	547 (22.2)	< 0.001^b^
500–810	486 (19.7)	464 (18.8)	514 (20.9)	440 (17.9)	413 (16.8)	
812–1125	561 (22.8)	497 (20.2)	491 (19.9)	483 (19.6)	426 (17.3)	
1129–1633	536 (21.7)	522 (21.2)	480 (19.5)	501 (20.3)	463 (18.8)	
> 1635	434 (17.6)	456 (18.5)	432 (17.5)	488 (19.8)	616 (25.0)	
*At least one transport trip reported, by mode of transport*—*n (%)*						
Walking	390 (15.8)	528 (21.4)	655 (26.6)	717 (29.1)	1068 (43.3)	< 0.001^c^
Cycling	38 (1.5)	36 (1.5)	51 (2.1)	82 (3.3)	136 (5.5)	< 0.001^c^
Public transport	64 (2.6)	144 (5.8)	238 (9.7)	282 (11.4)	459 (18.6)	< 0.001^c^
Private vehicle	2331 (94.6)	2253 (91.4)	2143 (86.9)	2085 (84.6)	1850 (75.1)	< 0.001^c^

Tests: ^a^Linear model (trend); ^b^Chi squared; ^c^Generalised linear model (trend); ^d^Linear model (square root transformed; trend); ^e^Survey day of administration was included as separate days in statistical models, but for conciseness summarised here as week or weekend day

Demographic differences relating to ULI quintile membership were observed. Most participants reported having access to a vehicle (96.7%); however, no vehicle access was more common for participants in higher ULI quintiles (*p* < 0.001). Participants in lower ULI quintiles were on average less likely to live in a single person household and more likely to have children (*p* < 0.001). A non-linear relationship was observed between liveability and income: those in the lowest and highest income brackets were similarly likely to live in a location of highest ULI, and more so than those in the middle-income bracket; those in the middle-income bracket were more likely to live in a low ULI quintile location compared with those in the highest and lowest income brackets.

There was a positive linear (unadjusted) association between the number of participants reporting taking at least one walking trip and ULI quintile membership (*p* < 0.001). Similar patterns were observed for use of public transport and for cycling. Conversely, those residing in more liveable areas had a reduced likelihood of travelling by private vehicle (*p* < 0.001).

This pattern was confirmed by the adjusted multilevel analysis as shown in Table 5 for walking for transport; columns represent estimates obtained using a progressively larger spatial aggregation of data for the indicators; and all indicators were included separately in each model. Full results including covariates for the hierarchical model predicting trips usage each transport mode with regard to the ULI are presented in Additional file 2.

**Table 5:** Covariate-adjusted estimates for change in odds ratio of taking a transport trip per unit increase in liveability (ULI) and sub-domains indicators (italics), with 95% Credible Intervals (CrI)

	Residential address $\widehat{AOR}$ (95% CrI)	Mesh Block $\widehat{AOR}$ (95% CrI)	SA1 $\widehat{AOR}$ (95% CrI)
Walking			
ULI	1.13 (1.12, 1.15)	1.14 (1.12, 1.16)	1.15 (1.13, 1.17)
ULI including air quality*	1.12 (1.09, 1.15)	1.13 (1.10, 1.16)	1.14 (1.11, 1.17)
*Walkability*	1.07 (1.07, 1.08)	1.07 (1.07, 1.08)	1.08 (1.07, 1.08)
*Social Infrastructure*	1.08 (1.07, 1.09)	1.08 (1.07, 1.09)	1.08 (1.07, 1.09)
*Public transport access meets policy (%)*	1.05 (1.04, 1.06)	1.06 (1.05, 1.07)	1.08 (1.07, 1.09)
*Large park access (%)*	1.00 (0.99, 1.01)	0.99 (0.98, 1.00)	1.00 (0.98, 1.01)
*Air quality*	–	0.94 (0.93, 0.94)	0.93 (0.93, 0.94)
*Affordable housing*	–	–	1.01 (1.00, 1.02)
*Local work opportunities*	–	–	1.00 (0.99, 1.01)
Public transport			
ULI	1.18 (1.15, 1.22)	1.19 (1.16, 1.22)	1.20 (1.16, 1.23)
ULI including air quality*	1.15 (1.11, 1.19)	1.15 (1.11, 1.20)	1.16 (1.11, 1.21)
*Walkability*	1.10 (1.10, 1.11)	1.10 (1.09, 1.11)	1.10 (1.09, 1.11)
*Social Infrastructure*	1.11 (1.10, 1.13)	1.11 (1.10, 1.13)	1.12 (1.10, 1.13)
*Public transport access meets* *policy (%)*	1.09 (1.07, 1.11)	1.11 (1.09, 1.13)	1.14 (1.12, 1.16)
*Large park access (%)*	0.99 (0.98, 1.00)	0.98 (0.97, 1.00)	0.99 (0.97, 1.00)
*Air quality*	–	0.90 (0.89, 0.91)	0.90 (0.89, 0.91)
*Affordable housing*	–	–	1.01 (1.00, 1.02)
*Local work opportunities*	–	–	0.97 (0.96, 0.98)
Cycling			
ULI	1.15 (1.11, 1.20)	1.16 (1.11, 1.22)	1.18 (1.13, 1.24)
ULI including air quality*	1.10 (1.04, 1.17)	1.11 (1.04, 1.18)	1.13 (1.06, 1.21)
*Walkability*	1.10 (1.08, 1.12)	1.10 (1.08, 1.12)	1.10 (1.08, 1.12)
*Social Infrastructure*	1.10 (1.08, 1.13)	1.10 (1.08, 1.13)	1.11 (1.08, 1.14)
*Public transport access meets* *policy (%)*	1.06 (1.03, 1.08)	1.08 (1.05, 1.11)	1.11 (1.08, 1.15)
*Large park access (%)*	1.00 (0.98, 1.02)	0.99 (0.97, 1.02)	1.00 (0.97, 1.02)
*Air quality*	–	0.91 (0.88, 0.92)	0.90 (0.88, 0.92)
*Affordable housing*	–	–	1.01 (0.98, 1.03)
*Local work opportunities*	–	–	0.97 (0.95, 0.99)
Driving			
ULI	0.85 (0.83, 0.87)	0.84 (0.82, 0.86)	0.84 (0.81, 0.86)
ULI including air quality*	0.88 (0.85, 0.91)	0.87 (0.84, 0.90)	0.86 (0.83, 0.89)
*Walkability*	0.91 (0.90, 0.92)	0.91 (0.90, 0.92)	0.91 (0.89, 0.91)
*Social Infrastructure*	0.90 (0.89, 0.91)	0.90 (0.90, 0.91)	0.90 (0.89, 0.91)
*Public transport access meets* *policy (%)*	0.94 (0.92, 0.95)	0.92 (0.90, 0.93)	0.90 (0.88, 0.91)
*Large park access (%)*	1.02 (1.01, 1.03)	1.03 (1.01, 1.04)	1.03 (1.01, 1.04)
*Air quality*	–	1.10 (1.09, 1.11)	1.10 (1.09, 1.11)
*Affordable housing*	–	–	0.99 (0.98, 1.00)
*Local work opportunities*	–	–	1.02 (1.00, 1.03)

Results are for separate models for each outcome-exposure of interest-scale combination, with adjustment for age, gender, household type and income, car ownership, employment and day of the week. Indicators based on larger aggregate data sources were identical at smaller aggregations or address points and so are omitted (–) in these cases

* Sensitivity analysis

The ULI was positively associated with walking, with an adjusted odds ratio (AOR) of 1.13(95% Credible Interval 1.12,1.15); that is, for each unit increase in the ULI score, the odds of walking for transport was estimated to increase by 13% (95% CrI 12% to 15%).

When modelled separately, three of the sub-domains of urban liveability were also significantly and positively associated with walking for transport: walkability AOR 1.07 (95% CrI 1.07, 1.08), social infrastructure AOR 1.08 (95% CrI 1.07, 1.09), and public transport AOR 1.05 (95% CrI 1.04, 1.06).

Conversely, the ULI and three of its sub-domains were significantly and negatively associated with travelling by private vehicle: ULI AOR 0.85 (95% CrI 0.83, 0.87), walkability AOR 0.91 (95% CrI 0.90, 0.92), social infrastructure AOR 0.90 (95% CrI 0.89, 0.91), and public transport AOR 0.94 (95% CrI 0.92, 0.95). These results demonstrate neighbourhoods that are designed to encourage walkability and active lifestyles are associated with increased uptake of active modes of transport including public transport, and decreased motor vehicle reliance.

**Fig. 5:**
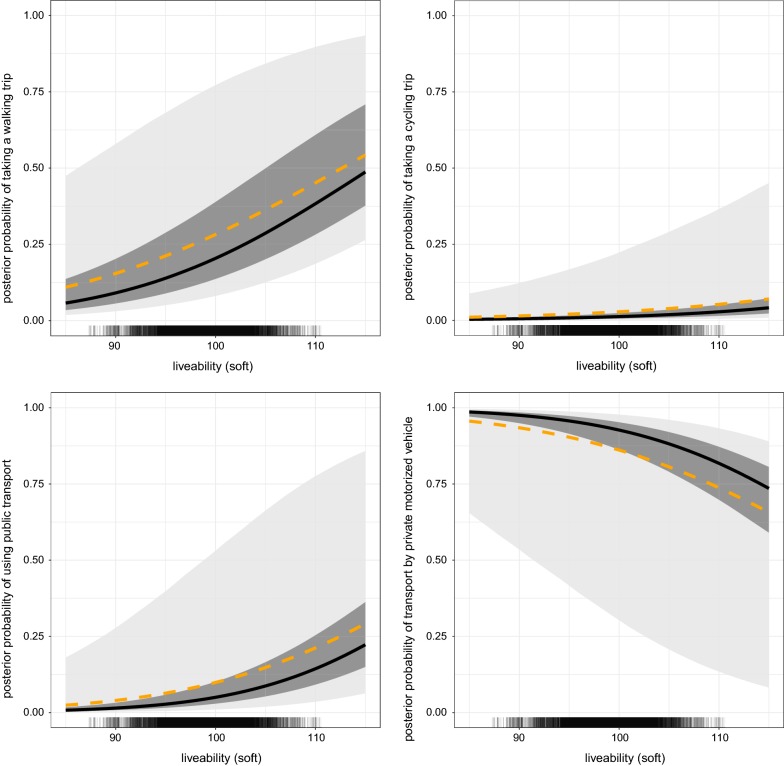
Posterior predicted probability of travel mode by liveability, adjusted for socio-demographic and area level effects. The dashed line represents the population-averaged probability; the solid line represents the median posterior predicted probability; shaded areas respectively represent the predicted probabilities for 50% (darker region), and 95% of the participants (lighter region)

We estimated the probability of taking a trip for the median person by each of the four modes considered (Fig. 5). As personal circumstances and preferences have a substantial role, we also plotted the influence of ULI on a ‘low’ and ‘high’ physically active person (corresponding to the 25th and 75th percentile of the posterior predictive distribution).The positive association between liveability and probability of walking for transport, in particular, was visibly appreciable. However significant variability between people yielded a wide distribution of posterior probability. This variation was influenced by covariates with effects of varying magnitude, and considerable variability between households.

The population-averaged probability of walking can be interpreted as the percentage of people that will walk for transport, and was notably higher than the probability of the median person. This demonstrates that, whilst our model predicts that living in more liveable areas is associated with increased probability of taking a walking trip, the magnitude of this increased probability depends upon individuals’ (measured) demographic factors and (unmeasured) personal preferences.

### Sensitivity analysis

#### Air quality

We refitted the hierarchical logistic regression models to an alternative version of the ULI with the addition of the air quality indicator (that is, reverse scaled NO_2_ air pollution predicted at the Mesh Block level for 2011). VISTA participant demographic characteristics summarised by liveability quintile when calculated with the inclusion of air quality are presented in Table 6.

**Table 6:** Summaries of covariates and outcomes of retained VISTA12 participants by hard threshold liveability score quintile (including air quality)

	ULI quintiles, including air quality					*p* value
	87.3, 96.0	96.0, 98.3	98.3, 100.1	100.1, 102.2	102.2, 110.6	
*N*	2465	2464	2465	2465	2464	
*Age*						
Continuous—median [IQR]	47 [24.0]	46 [24.0]	46 [25.0]	45 [25.0]	46 [26.0]	0.972^a^
Group—n (%)						< 0.010^b^
18–29	431 (17.5)	429 (17.4)	447 (18.1)	434 (17.6)	434 (17.6)	
30–49	935 (37.9)	1011 (41.0)	987 (40.0)	1016 (41.2)	938 (38.1)	
50–64	745 (30.2)	663 (26.9)	652 (26.5)	613 (24.9)	692 (28.1)	
65 and over	354 (14.4)	361 (14.7)	379 (15.4)	402 (16.3)	400 (16.2)	
*Sex*						0.528^c^
Male	1199 (48.6)	1227 (49.8)	1190 (48.3)	1203 (48.8)	1186 (48.1)	
Female	1266 (51.4)	1237 (50.2)	1275 (51.7)	1262 (51.2)	1278 (51.9)	
*Household structure*						< 0.001^b^
Lone person	176 (7.1)	225 (9.1)	296 (12.0)	294 (11.9)	392 (15.9)	
With children	774 (31.4)	840 (34.1)	679 (27.5)	695 (28.2)	610 (24.8)	
Without children	1515 (61.5)	1399 (56.8)	1490 (60.4)	1476 (59.9)	1462 (59.3)	
*Employed*—*n (%)*						< 0.001^c^
Yes	1810 (73.4)	1788 (72.6)	1745 (70.8)	1738 (70.5)	1667 (67.7)	
*Vehicle access*—*n (%)*						< 0.001^c^
No	23 (0.9)	64 (2.6)	105 (4.3)	86 (3.5)	139 (5.6)	
*Survey administration day of week*^*e*^—*n (%)*						< 0.004^b^
Weekday	1922 (78.0)	1880 (76.3)	1847 (74.9)	1836 (74.5)	1900 (77.1)	
Weekend	543 (22.0)	584 (23.7)	618 (25.1)	629 (25.5)	564 (22.9)	
*Equivalised household income (pre*-*tax weekly $AUD)*						
Continuous—median [IQR]	972.2 [803.6]	985.7 [878.1]	980 [905.6]	964.3 [906.2]	933.3 [940.0]	< 0.008^d^
Quintiles—n (%)						< 0.001^b^
< 500	438 (17.8)	482 (19.6)	569 (23.1)	532 (21.6)	599 (24.3)	
500–810	480 (19.5)	476 (19.3)	452 (18.3)	465 (18.9)	444 (18.0)	
812–1125	586 (23.8)	487 (19.8)	428 (17.4)	510 (20.7)	447 (18.1)	
1129–1633	521 (21.1)	539 (21.9)	532 (21.6)	437 (17.7)	473 (19.2)	
> 1635	440 (17.9)	480 (19.5)	484 (19.6)	521 (21.1)	501 (20.3)	
*At least one transport trip reported, by mode of transport*—*n (%)*						
Walking	416 (16.9)	603 (24.5)	699 (28.4)	757 (30.7)	883 (35.8)	< 0.001^c^
Cycling	43 (1.7)	57 (2.3)	72 (2.9)	80 (3.2)	91 (3.7)	< 0.001^c^
Public transport	78 (3.2)	213 (8.6)	267 (10.8)	313 (12.7)	316 (12.8)	< 0.001^c^
Driving	2304 (93.5)	2197 (89.2)	2074 (84.1)	2082 (84.5)	2005 (81.4)	< 0.001^c^

Tests: ^a^lm(trend); ^b^Chi-squared; ^c^glm (trend); ^d^lm(sqrt) (trend); ^e^survey day of administration was included as separate days in statistical models, but for conciseness summarised here as week or weekend day

The model including air quality has a lower expected log predictive density estimate (− 6306.8 vs. − 6277.8), suggestive of a worse fit to the data than the model with air quality excluded. Further, the effect size estimates for association between the ULI and travel mode choice were attenuated, and credible interval bounds were broadened for each mode choice (Table 5). This result is consistent with the negative association between air quality and walking for transport (AOR 0.94, 95% CrI 0.93, 0.94), with similar results for public transport use and cycling. Nevertheless, the ULI remained positively and significantly associated with active travel modes (walking, cycling and public transport), and negatively associated with private motor vehicle use.

Noting that the two approaches to ULI construction—excluding and including air quality—do not have the same distribution, this result may be better represented by considering the estimated effect per interquartile range. For the ULI excluding air quality (IQR 6.64), the AOR per IQR increment for taking at least one walking trip was AOR 2.29 (95% CrI 2.06, 2.56); while for the ULI including air quality (IQR 4.98) the AOR was 1.75 (95% CrI 1.56, 1.96). This reveals a substantial reduction in estimated effect size for the model including the air quality indicator.

#### Aggregation

To investigate the effect of aggregation, we refitted the model substituting Mesh Block and SA1 averages for the true address level indicators (Table 5). For some indicators, aggregation had little or no influence, however, for public transport the estimate of the effect size was biased upwards: when using SA1 level aggregation instead of address level data the increased odds of walking per unit of public transport access changed from 5% to 8%; the estimated percentage increase in odds of walking for transport for a unit change in ULI also increased approximately 2% in magnitude to 15% (95% CrI 13%, 17%).

#### Threshold method

The final conditionally scaled and normalised ULI estimates, calculated using soft thresholds, showed an increased range and reduced interquartile range compared with alternate hard threshold versions (Table 7). Comparison of the 25th, 50th (median) and 75th percentile positions of the distribution for the destination access-related indicators across hard and soft threshold versions highlight the value of soft thresholds. For example, based on the raw version of the hard threshold public transport access indicator, an average of 68.4% of residential addresses have access to public transport within recommended walking distance; using the soft threshold indicator we see that, in general, effective access scores range between 32.3% and 95.5%, with a median of 81.9% and reduced mean and standard deviation of $64.5 \pm 36.1$. These results support the intuitive notion that depending on where a person lives, using hard or soft threshold approaches to destination access will meaningfully impact the estimate for a given indicator and overall liveability for that particular address.

**Table 7:** Summary of address-level hard- threshold indicator and ULI (in italics) distribution

	Hard threshold ULI					
	Mean ± SD	25th p.	Median	75th p.	Min.	Max.
*Raw indicators*						
Walkability†	0.0 ± 2.4	− 1.3	− 0.1	1.0	− 7.4	13.4
Daily Living (/3)†	1.8 ± 1.0	1.0	2.0	3.0	0.0	3.0
Dwellings per Ha	14.1 ± 5.8	11.1	13.3	15.7	0.0	52.1
3 + way street connections per km^2^	70.2 ± 20.5	60.3	67.4	76.2	2.0	201.2
Social infrastructure mix (/16)†	7.1 ± 3.7	4.0	7.0	10.0	0.0	16.0
Public transport access meets policy (%)†	68.4 ± 46.5	0.0	100.0	100.0	0.0	100.0
Large park access (%)†	49.0 ± 50.0	0.0	0.0	100.0	0.0	100.0
Air pollution (predicted NO_2_ ppb.)	8.9 ± 2.6	7.1	8.7	0.3	3.6	34.9
Affordable housing (SA1 %)	11.4 ± 8.9	5.4	10.5	16.2	0.0	100.0
Local work opportunities (SA2 %)	26.6 ± 11.2	19.4	24.4	29.7	10.0	68.7
*Conditionally scaled (compressed outliers)*						
Walkability†	−0.1 ± 2.1	− 1.3	− 0.1	1.0	− 7.2	7.2
Daily Living (/3)†	1.8 ± 1.0	1.0	2.0	3.0	0.0	3.0
Dwellings per Ha	13.9 ± 4.9	11.1	13.3	15.7	0.0	31.4
3 + way street connections per km^2^	69.2 ± 17.0	60.3	67.4	76.2	8.7	131.6
Social infrastructure mix (/16)†	7.1 ± 3.7	4.0	7.0	10.0	0.0	16.0
Public transport access meets policy (%)†	68.4 ± 46.5	0.0	100.0	100.0	0.0	100.0
Large park access (%)†	49.0 ± 50.0	0.0	0.0	100.0	0.0	100.0
Air pollution (predicted NO_2_ ppb.)	8.8 ± 2.3	7.1	8.7	10.3	3.6	16.6
Affordable housing (SA1%)	11.1 ± 8.2	5.4	10.5	16.2	0.0	38.2
Local work opportunities (SA2%)	26.2 ± 10.2	19.4	24.4	29.7	10.0	60.1
*Normalised, conditionally scaled Indicators, with composite index*						
*ULI*†	*99.2 ± 4.9*	*95.9*	*99.4*	*102.5*	*83.1*	*118.9*
ULI including air quality*†	*99.1 ± 3.8*	*96.6*	*99.2*	*101.7*	84.9	*112.6*
Walkability	100.0 ± 10.0	94.1	99.7	104.9	66.6	134.2
Daily Living (/3)†	100.0 ± 10.0	92.3	101.9	111.5	82.7	111.5
Dwellings per Ha	100.0 ± 10.0	94.4	98.9	103.8	71.7	135.9
3 + way street connections per km^2^	100.0 ± 10.0	94.8	99.0	104.1	64.3	136.8
Social infrastructure mix (/16)†	100.0 ± 10.0	91.6	99.8	108.0	80.7	124.3
Public transport access meets policy (%)†	100.0 ± 10.0	85.3	106.8	106.8	85.3	106.8
Large park access (%)†	100.0 ± 10.0	90.2	90.2	110.2	90.2	110.2
Air quality	100.0 ± 10.0	93.5	100.5	107.4	66.2	122.7
Affordable housing (SA1 %)	100.0 ± 10.0	93.0	99.3	106.3	86.4	133.2
Local work opportunities (SA2 %)	100.0 ± 10.0	93.3	98.2	103.4	84.1	133.2

Constituent sub-indicators contributing to walkability score are included for reference purposes

* Sensitivity analysis

^†^ Indicators or composite indices with values which may be impacted by choice of hard- or soft- destination threshold

**Fig. 6:**
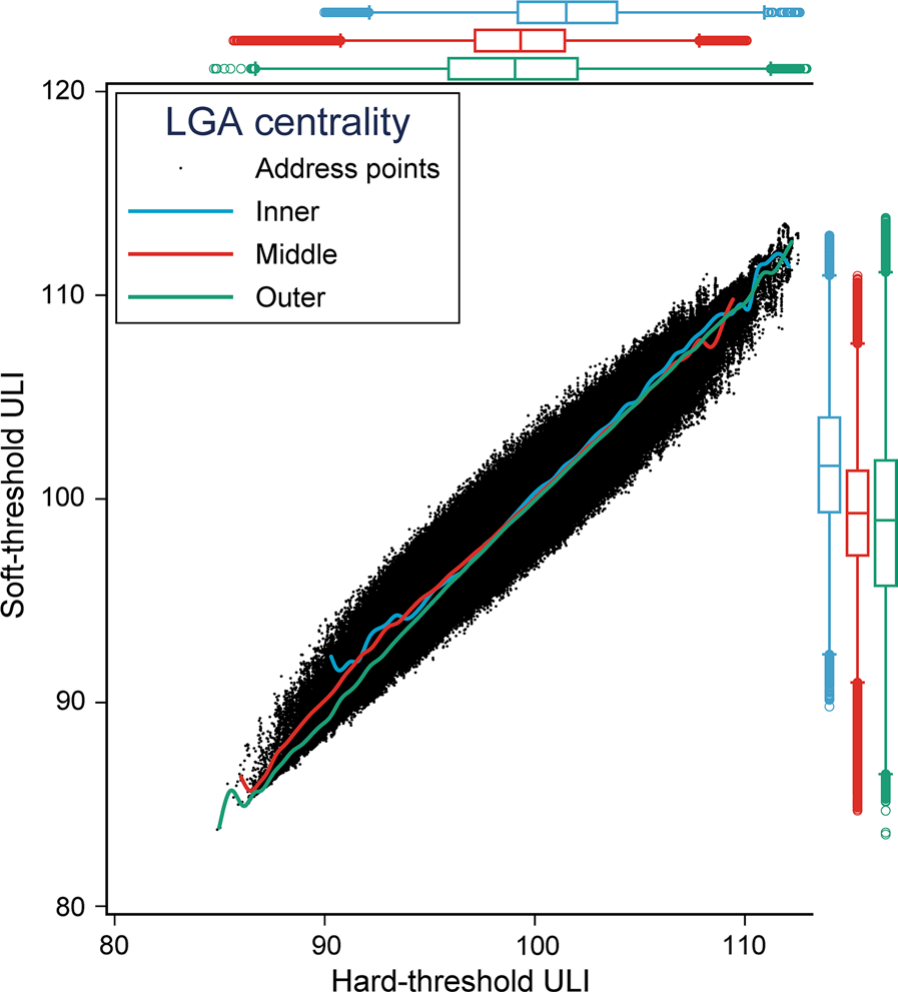
Scatterplot of hard- and soft-threshold versions of the ULI with marginal distribution boxplots and cross-median fitted splines of trend by LGA centrality: Inner (blue), Middle (red) and Outer (green)

The bivariate distribution of hard- and soft-threshold liveability, stratified by centrality of LGA is presented in Fig. 6. Addresses located in inner city LGAs had an average soft ULI score of $101.6 \pm 3.32$, which evaluated separately using a *t* test was 2.6 points higher ($p < 0.001$) than that of the combined average of middle ($99.2 \pm 3.16$) and outer ($98.8 \pm 4.29$) LGAs. A similar trend observed using hard thresholds.

The marginal averages of the ULI calculated using the two approaches to destination measurement were similar regardless of the air quality inclusion, however the soft threshold ULI had a small increase in standard deviation (Table 6). The inclusion of air quality decreased variation in both ULI versions: excluding air quality, hard threshold ULI $99.2 \pm 4.9$ and soft threshold ULI $99.2 \pm 5.0$; including air quality, hard threshold ULI $99.1 \pm 3.8$ and soft threshold ULI $99.2 \pm 3.9$.

The hard and soft thresholds of the ULI (with and without air quality) and their respective equivalent ULIs were highly correlated with each other (population correlation $\rho = 0.97,$ Table 8). The lower left and upper right triangle segments of Table 8 respectively detail correlation between indicators and their composites for the hard and soft threshold ULI versions; the matrix diagonal presents the correlation between hard and soft threshold ULI versions of the equivalent indicator. Air quality was negatively correlated with both hard and softthreshold walkability (respectively, $\rho =$ − 0.65 and $\rho =$ − 0.67).

**Table 8:**
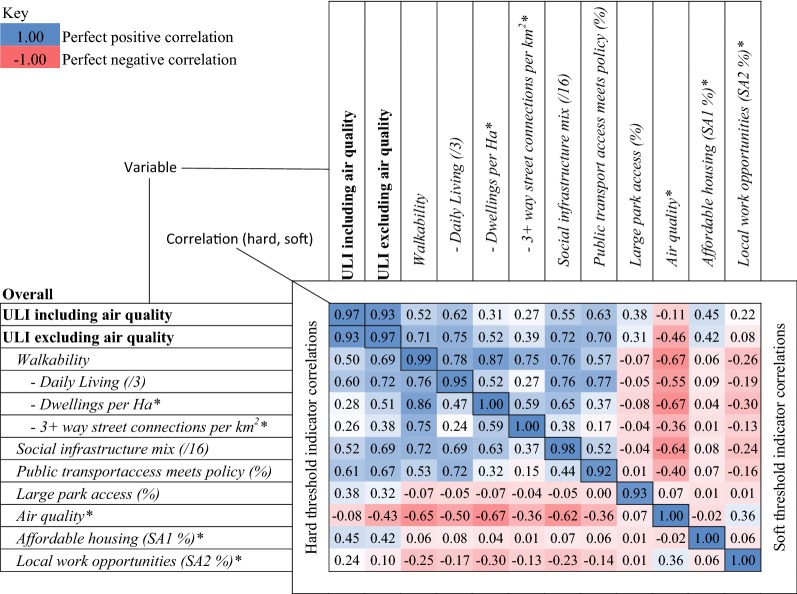
Correlation matrix for hard- and soft-threshold sets of indicators, the ULI including and excluding air quality

* These variables do not employ hard- or soft- thresholds for distance; hence, perfect correlation between the hard and soft ULI

## Discussion

There is growing recognition internationally that more ‘liveable’ neighbourhoods and cities positively impact quality of life and the health and wellbeing of residents. However, to date, the field has been hampered by methodological limitations and lack of measurement. This has made it difficult to draw robust conclusions about the combined health effects of liveability attributes, and therefore to inform and monitor policy recommendations for creating liveable cities that enhance health and reduce inequities. We sought to respond to these limitations by creating an evidence-informed, policy-relevant tool that measures the distribution of urban liveability within cities. Importantly, the ULI enables interrogation of how liveability is related to health and wellbeing, and for whom. Such evidence can be used to inform policies and targeted interventions to plan for cities that optimise health for all.

### Population-level association between liveability and travel behaviour

Transport mode choice was associated with ULI both in univariate analysis and in the hierarchical logistic regression model. Our model predicts doubling to tripling the incidence of walking, public transport usage and cycling in communities with the highest compared with the lowest ULI scores. This suggests that if we designed more liveable communities, at the population level, there would be an almost-linear overall increase in the number of people walking for transport on any given day, offset by a decrease in people travelling by private motor vehicles.

Estimated effects of such magnitude seem optimistic and, of course, multiple other factors may have contributed to these estimates. In particular, the models also indicate that there is large variation in people’s preferences: people with stronger preferences towards active travel are more likely to move into more liveable neighbourhoods, if they can afford to do so. Indeed, previous studies have suggested a latent demand for more walkable communities, with the majority of people living in low walkable environments preferring instead to live in areas where they could walk to local amenities [52, 53]. However, other factors influence people’s choice of neighbourhoods, with the most important factors being housing affordability [54]. As such, it seems unlikely that neighbourhood selection as a common cause can explain all of the observed association; the influence of neighbourhood selection is not necessarily greater than that of built environment on behaviour [55].

At least some of the observed effects are likely due to neighbourhood selection. Future studies could consider measuring and adjusting for personal preferences and length of time in neighbourhood. In this study, we used a cross sectional state government transport survey data set, which did not include preferences; this makes it challenging to assess such conflicting effects. Overcoming such limitations requires both travel survey data as well as preferences to be collected, in a longitudinal study. This could be in the form of (say) a natural experiment [56, 57]. Testing the ULI within the context of a natural experiment would enable the independent effect of urban liveability to be rigorously established by disentangling it from the differing demographic, personal preference, and previous behaviour profiles of residents.

An important consideration when evaluating the association between a composite indicator of environmental exposure such as the ULI and a particular health related outcome like travel mode choice, is that some of the component indicators will be more or less strongly associated with the outcome than others; by considering the overall association for the ULI, the associations of the underlying indicators may be masked. For this reason, we analysed the associations with each indicator in addition to the ULI using separate models. As a summary measure, the ULI is best considered as a lens through which to consider liveability; presented with a particular score, it invites users to interrogate how the various domains of liveability contribute to this estimate. This is readily achieved through interactive mapping, where a choropleth of area level liveability distribution may be clicked to display a break-down of the indicators giving rise to the ULI for a particular area.

**Fig. 7:**
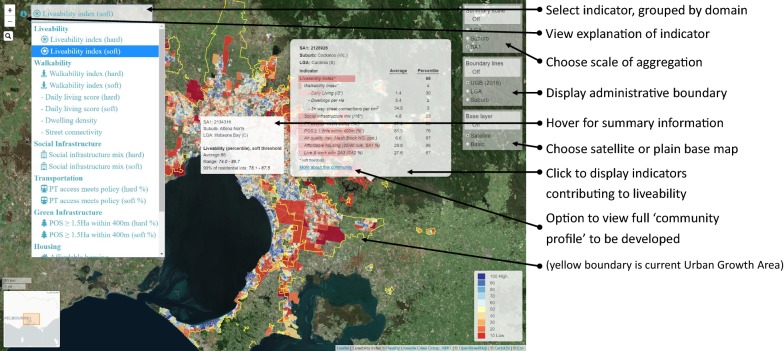
Annotated screenshot of the pilot Urban Liveability Index in a prototype urban indicators observatory, currently under development by the Healthy Liveable Cities group at RMIT University

A prototype online interactive dashboard for the pilot urban liveability index is under development by our group at the time of writing (Fig. 7). A user can display a choropleth visualisation for a chosen indicator at a selected scale and use hover or click behaviour to explore different aspects of summary information, such as the usual range of values associated with addresses within a particular area; the mix of indicators which contribute to a liveability estimate; and various administrative boundaries. The maps included in the Additional file 1 animation were also derived from a prototype of such an approach.

### Assessing the relationship between urban liveability and air quality

As noted earlier, in our original conceptualisation of liveability, air quality was considered an outcome of effective city planning decision-making [7, 9] and was not scoped for inclusion in the liveability index [10]. However, there are growing global concerns about the health impacts of air quality. With approximately 5% of mortality in 2017 estimated to be attributable to ambient particulate matter pollution, air quality is considered the 8th leading risk factor for death globally [58]. Hence, we conducted a sensitivity analysis to evaluate the impact of creation and testing of an alternate ULI including air quality. Comparing the modelled association between active transport and the ULI with and without air quality, we found that the ULI without air quality resulted in a better fit. This result is not surprising, as there is no a priori reason to expect a strong direct effect of air quality on travel mode choice. Moreover, air quality was negatively correlated with most other sub-indicators, including walkability; a relationship noted in recent international literature [59, 60].

The negative correlation between air quality and active transport behaviour, as well as the negative correlation with other indicators, indicates that people are more likely to walk and cycle in areas where air pollution is higher. This is not surprising because those areas have more amenities, but nevertheless is a concern. Generally, highly walkable areas attract both pedestrians and traffic because there are more destinations available.

Air pollution concentrations in Melbourne are relatively low by global standards. However, ‘safe’ long-term exposure thresholds have not been identified for most air pollutants. There is a clear tension here: a major challenge to policy-makers is how to design highly walkable mixed-use areas with good access to shops and services, while minimising exposure to vehicular traffic and consequential, traffic-related air pollution. Nevertheless, evidence suggests that the health benefits gained from using active transport modes, such as walking and cycling, outweigh the risks when compared to sedentary modes of transport such as driving [61, 62]. Nevertheless, our results suggest there would be benefit to exploring how streets with shops and services could be pedestrianised to avoid exposing pedestrians and local residents to transport-related air pollution.

### Analysis using aggregated liveability summaries

Refitting of the travel behaviour models using data aggregated at progressively larger scales increased the magnitude of effect size estimates for the association between the ULI (all versions); odds ratios increased at each larger scale for walking and cycling and reduced for private motor vehicle. It is well known that the use of aggregated data may lead to biased estimates [63]. In this study, we observed estimated effect sizes increasing in magnitude with higher levels of aggregation for the association between public transport access and the probability of making a walking trip. This upward bias was apparent even though our aggregation in our study was based on complete data on individual-level indicators. However, area-level estimates are sometimes based upon a small sample or singleton (e.g. population weighted centroid) of representative points within each area; this would lead to further measurement error, and impact the bias and precision of estimates obtained from a regression analysis or other statistical model. In cases where the accurate representation of individual liveability is a concern and access to sufficiently granular data is achievable, we recommend the calculation of individual-level liveability estimates.

### Destination access measurement and threshold methods

The use of soft threshold destination access indicators was a novel method developed for this project, designed to depict a more nuanced picture of the achievement of policy standards, than that derived using hard thresholds. In this study we found that the hard and soft threshold ULI versions were highly correlated overall and bore similar relationships with their constituent indicators. However, we identified socio-spatial differences. For example, inner- and middle-regions of relatively greater disadvantage tended to receive a larger increase in liveability rank than those in outer regions when estimating liveability using soft instead of hardthresholds for destination access. This finding highlights the importance of the selected threshold for indicator measurement, and subsequent impact on representation of the spatial distribution of liveability, alongside the importance of socio-economic context in testing appropriate methods. By allowing residential addresses to have access scores of ‘some’ (soft thresholds) rather than ‘none’ (hard thresholds), we could better identify and locate population inequities. This is highly relevant to policy-makers seeking practical ways to reduce spatial inequities across a city.

A limitation of a soft threshold indicator of destination access is that the area-aggregated mean no longer represents the proportion of those who have access. Hard thresholds, which are readily interpreted as proportions when aggregated, are of clear use for those seeking to communicate and measure adherence to urban policy. However, the softthreshold indicators may be a more useful measure for assessing *effective* access, and for other purposes such as mapping relative inequities, identifying locations for interventions, or for statistical analysis purposes such as regression modelling and simulation. Furthermore, once incorporated into a composite measure, the interpretative benefit from use of hard threshold indicators is lost. As such, the more detailed measurement of access which soft threshold indicators impart to the ULI composite measure is appreciable.

There is a broad literature on calculation of individual level measures which can be used to estimate accessibility given limits or weights on distance, trip time, proximity and utility of multiple transport choices, and accounting for attractiveness of destinations; these are alternative options which may be considered [64, 65]. In many instances it may be desirable to consider the influence of proximity to an amenity on a health outcome using distance alone. However the present index has evolved out of research concerned with policy based indicators which recommend access to different kinds of destinations within specific distances; that is, liveability with regard to meeting evidence-based policy recommendations, where available. Had distance alone been used, all kinds of destinations for which proximity was considered would have been treated equally important; weighting could be introduced to re-introduce a sense of relative importance, however this would again result in a measure even further abstracted from existing policy. In the present study we adopted a policy-informed threshold distance approach as used on grounds of parsimony and relevance to our study aim of providing a policy relevant liveability index [66]. The present study introduces and provides some preliminary consideration of the validity of the soft threshold approach, contrasted with the hard threshold approach; future studies may expand on this, with comparisons to other measures.

### Liveability and centrality of Local Government Area

ULI estimates were higher on average for addresses located within inner-Melbourne than for those in middle and outer regions (Additional file 1: Video S1). This finding reflects acknowledged inequities in the distribution of amenities, employment opportunities and transport infrastructure access, which policy initiatives such as the Victorian Governments Plan Melbourne 2017–2050 seek to address [5, 67]. The rapid growth of cities and associated low density development on the urban fringe perpetuates inequities in outer suburban areas. While housing may be more affordable, these areas may not facilitate affordable living because they lack proximal access to public transport and local amenities. Our research indicates that densities of at least 30 dwellings per hectare are required to create neighbourhoods that encourage active modes and public transport use and decrease driving [68]. This is because sufficient density is required to support local economics and to make public transport more viable.

### Indicator choice

The ULI was comprised of a modest selection of liveability indicators representing core domains of liveability and the social determinants of health, serving as a simple but readily calculable and useful model of the combined influence of the social determinant of health and wellbeing. Future work can expand on this. Our time point of 2011 was chosen due to that being the most recent census available at the commencement of this project. However, the landscape of available data has changed much in recent years: General Transit Feed Specification (GTFS) data, has become broadly available for Australia and in some international settings, allows for a standardised approach to recording public transport service frequency. This provides a much more detailed record of a place’s potential for public transport than the presence of, say, a bus stop that may or may not be serviced. In Australia, the 2016 census allows for consideration of small area average times for journey to workplace by employment sector; this is a rich source of data for development of spatial indicators of employment potential. Walkability could be disaggregated prior to inclusion in the ULI giving its constituent aspects more weight in the final composite, and nuanced with more local environment indicators: slope, temperature, humidity, quality and accessibility of footpaths are important aspects future work could consider, given data availability. Our suite of indicators did not capture social and cultural diversity, nor local mix of housing options or access to digital infrastructure; we acknowledge these as important aspects of liveability. Our green infrastructure measure of large park access only evaluated quality in terms of size. This is relevant from a policy perspective and was a pragmatic option given our data limitations; however, lived experience and choice to interact or not with a space is may be expected to be influenced by other aspects includingaesthetics, quality and appropriateness of service provision given population demand. These are just some of the avenues along which future work may expand on the ULI concept.

### Indicator outliers and the Urban Liveability Index

The ULI method is based on approach which penalises inconsistency across indicators [48]. However, when developing our approach we considered that the potential penalty arising from variation in a set of indicator results could be disproportionately large relative to the mean of the indicators. An example would be for a residential lot in an area performing exceedingly well on all but one indicator, having extremely poor performance: the otherwise excellent performance could be disproportionately negated impacting the ULI’s face validity if the penalty were not constrained. We therefore implemented an approach whereby outliers were still penalised, but the maximum penalty to the overall ULI result was limited such that overall good performance was still rewarded. We considered this approach to provide improved face validity of the composite results compared with not undertaking this transformation. Future studies could consider the impact of outlier treatment in more detail.

### ULI construction method choices

The ULI was developed as a result of considerable interest amongst local policy makers, about urban liveability and the cumulative influence of the underlying domains of liveability on health-enhancing behaviours of residents. It has also been designed to work in an interactive mapping application that can be used as a diagnostic tool by policy-makers to identify what interventions might enhance the living conditions of residents in different part of Melbourne. It is plausible that a simpler index would suffice, particularly in terms of predicting travel mode choice. For example, it could be that in some cities, density alone would be a sufficient proxy of the ULI. However, in some cities or areas within cities, high density development is simply ‘high rise sprawl’, because it is not accompanied by amenities that enhance the liveability of a city. In this sense, some caution is required. Nevertheless, this question warrants further investigation in terms of developing a more simple, parsimonious index that captures the essence of a liveable city. Given global interest in liveability, we believe this research is warranted.

We adopted the Mazziota–Pareto method for composite index construction following our review of the OECD guide to composite indicator construction and literature on developing composite indicators for wellbeing and sustainability. Factor analysis and principal component analysis (PCA) were options flagged for consideration due to their use for achieving a more parsimonious index through dimension reduction. However, a key driver of our choice of method was ability to meaningfully communicate the ULI—and performance of its underlying domains—to policy makers and planners who do not necessarily have backgrounds in statistical methodology. The ULI draws upon the established MPI-approach for calculation of composite indicators in wellbeing contexts, and its meaning when aggregated for an area is readily conveyable in plain language to a lay audience: it reflects an area’s relative performance across core aspects of liveability and incentivises balanced performance across these domains.

When constructing the ULI we were not only challenged to contemplate which objective exposures should be considered as measuring liveability, but also to determine their relative importance. Finding consensus amongst experts and stakeholders on weighting when constructing composite indicators has been noted as a fraught task [87]. Factor analysis and PCA are attractive in that they allow empirical derivation of such weights. However, such weighting will be contingent on the sample used to inform the analysis and so this weighting may not be generalisable to other contexts. In the preliminary stage of our development of the ULI we considered other methods such as the ‘Benefit of the Doubt’ approach which was explicitly designed to be used in policy contexts: sensitive comparisons between jurisdictions with differing contexts and priorities may require use of a weighting scheme that plays to the relative strengths of an area to achieve common agreement [87]. However, we considered that this endogenous approach to weighting could negate the combined importance of these domains; hence our choice of an approach that encourages a balanced, but well-performing score across all domains.

The ULI does not directly weight the indicators from which it is formed. However, the relative importance of each indicator in the ULI is influenced by the choice and form of indicators included: for example, by including the walkability index in its composite form as we did in this study each of its components (dwelling density, street connectivity, and land use mix) were afforded relatively less weight than had they been included separately as indicators in their own right. Likewise, were we to have included the social infrastructure mix sub-domains—’early years’, ‘education’, ‘community, culture and leisure’, ‘health and social services’ and ‘sport and recreation’—as separate indicators this would have added more weight to the importance of proximity to a well serviced community. We plan to investigate these options further in future development of the ULI. Other future studies may therefore wish to compare various approaches to index development.

### Future applications

The ULI scripted approach was designed with extension to future applications in mind, in particular to facilitate creation of a national liveability index for Australian cities. It is possible to extend the ULI to accommodate sub-group specific domain weighting profiles, recognising that even on average, not all indicators would be of equal importance to all groups of people. For example, one’s age, household composition, level of functional ability, or personal preferences could influence the relative importance of specific measures. An approach accounting for different ‘liveability profiles’ could be informed and developed through mixed methods studies with sub-populationsor through natural experiments, in order to estimate a demographically-nuanced ULI.

The ULI aligns with and supports a number of the UN Sustainable Development Goals (SDGs), including goals 3 ‘Good health and well-being for people’ and 11 ‘Sustainable cities and communities’ [3]. More ‘liveable’ cities are likely to encourage active forms of transport, and positively impact residents’ wellbeing, enhance resilience and sustainability, and reduce poverty [84]. Therefore, exploring whether it is feasible to extend this work to other city contexts and for use as a monitoring tool by UN Habitat to evaluate the New Urban Agenda [85, 86] is worthy of consideration. The indicators included in the ULI may be more or less relevant for different cities and relevant data may not always be readily available, particularly for cities located in rapidly developing countries. Future research of this nature might help to better address equity questions, such as ‘liveable for whom?’ [69].

A major opportunity in the face of population growth and rapid urbanisation, is to monitor changes in liveability across time. The geographic and temporal scope of this pilot project was limited for pragmatic reasons to Melbourne in 2011; a logical extension of this project will be to calculate estimates of ULI for other Australian capital cities and then monitor changes over time. This work has commenced with the development of national liveability indicators for Australian capital cities, aligned to both health outcomes and policy [70]. The next stage will involve developing a comparable ULI for these cities. More broadly, we are also consulting with regional and rural local governments to understand how the concept of liveability may be adapted to meet the distinct needs of populations residing in non-metropolitan locales. To be usable with longitudinal data, the ULI will need to be adapted to calculate changes for a particular locale over time. Three options are available: standardising the constituent indicators over all space–time points, or, with respect to the baseline observations, or, with respect to the final observations [71]. Similarly, multiple regions could be compared either with the overall standard, or one region could be assessed to the standard of a reference region [24].

Moving forward, it is envisaged the ULI could be useful in providing both exposure and outcome tools for researchers and other stakeholders, in particular urban planners who are attempting to create more health-promoting liveable cities. As an *exposure* measure, the ULI is designed to enable linkage with geocoded population health and social survey datasets. In this way, researchers could examine how the combination of different built environment attributes (related to the social determinants of health) relate to a range of health, wellbeing, social and economic outcomes as captured through population surveys (including linking with longitudinal data). The ULI could also be used to study inequities in access to social determinants of health, and associations with both healthy behaviours and health outcomes may be compared with those of simpler or more focusedmeasures such as the walkability index. In contrast to these, the ULI extends the research questions that can currently be asked of many built environment and health data sets by investigating the *cumulative* effects of integrated urban planning, which is likely to be of particular interest to city policy-makers. As an *outcome* measure, the ULI could be mapped and used to monitor cross-sectionally and longitudinally the liveability of a given neighbourhood or region, and to identify inequities in urban liveability between and within cities. In the Australian context, we have already begun identifying and testing liveability indicators and evaluating whether policies designed to create liveable cities are being delivered. This allows us to move beyond observational analysis to undertake natural experiments of city planning policies, and to enable domains of ‘liveability’ to be visualised across given study regions [70]. In this way, the ULI could be used to benchmark and monitor progress towards achieving local, state, or national policies that aim to create more liveable communities.

## Conclusion

The ULI was developed to study the impact of the distribution of accessibility and availability of liveability domains conceptualised to support health and wellbeing, by combining these into a policy-relevant and evidence-informed composite index. We found that living in more liveable areas with higher ULI scores was associated with higher levels of walking, cycling and public transport as travel modes, and lower levels of private motor vehicle use. These associations were in the expected direction, approximately linear and provide face validity of the ULI extending commonly used walkability measures and capturing broader built environment attributes with the potential to promote health. Although these results require further validation elsewhere, they suggest that creating more liveable communities has the potential to produce co-benefits for public health, the environment and managing traffic congestion [84].

The ULI allows the evaluation of integrated urban planning policies for liveable neighbourhoods and for the health impact of the integration of those policies to be assessed. Moreover, the mapping of the ULI enables the assessment of how well policies are being delivered within a study region of interest, and for whom. Specific policies that are (or are not) being delivered may be identified through disaggregation of the ULI into its sub-domains, enabling identification of opportunities for future targeted portfolio investment. Inequities in liveability across cities can be visualised and interrogated by state and local government policy makers, in order to devise appropriate interventions. In addition, the ULI can be used as an exposure measure in statistical modelling, thus providing a potentially powerful tool for assessing the upstream determinants of the delivery of the UN Sustainable Goals, alongside local urban planning policies.

## Additional files

**Additional file 1. Video S1.** Animated sequence allowing for comparison of choropleth maps of the spatial distribution of quartiles of the Urban Liveability Index with- and without air quality included, and the raw forms of each of its constituent indicators, with aggregation at the SA1 small area scale.

**Additional file 2.** Additional tables in presenting the full posterior log odds estimates and their exponentiated adjusted odds ratio forms for the main hierarchical models fitted with regard to the Urban Liveability Index.

## Data Availability

The data that support the findings of this study are or were available from ABS [72–75, 78], PSMA [42], DSE [76, 82], VEAC [77], Knibbs [80], Acxiom [81], and ACECQA [83]. However, restrictions apply to the availability of some of these data, which were used under license for the current study [42, 81] or are historical and no longer hosted in the same form online by the source institutions [76, 77, 82, 83]. Our final derived data source contains linked geocoded VISTA survey participant data, sourced under licence from the thenVictorian Department of Transport, Planning and Local Infrastructure; as such, public dissemination is subject to ethics approval, and the survey data itself should be obtained from the data custodian.
