# Supplementary material for: The Urban Liveability Index: developing a policy-relevant urban liveability composite measure and evaluating associations with transport mode choice
Source: Int J Health Geogr. 2019 Jun 11;18:14. doi: 10.1186/s12942-019-0178-8 (PMC6558748; doi:10.1186/s12942-019-0178-8)
Supplement: Supplementary file 2 — Additional file 2. Additional tables in presenting the full posterior log odds estimates and their exponentiated adjusted odds ratio forms for the main hierarchical models fitted with regard to the Urban Liveability Index. [file 12942_2019_178_MOESM2_ESM.pdf]

Additional Table 1. Posterior estimates including covariates and random effects for hierarchical model predicting the log odds of walking for transport. Estimates are on the log-odds scale to facilitate comparing the magnitude of effect between measured covariates and random effects. The exponentiated log odds estimates are presented as adjusted odds ratios in Additional Table 5.

**Covariates**	**mean**	**sd**	**Posterior distribution percentiles**				
			**2.5%**	**25%**	**50%**	**75%**	**97.5%**
**ULI**	0.13	0.01	0.11	0.12	0.13	0.13	0.14
Age group							
18 – 29 year old				*reference*			
30 – 49 year old	-0.15	0.09	-0.33	-0.22	-0.15	-0.09	0.03
50 – 64 year old	-0.27	0.09	-0.44	-0.33	-0.27	-0.21	-0.09
65 or older	-0.26	0.12	-0.48	-0.34	-0.26	-0.18	-0.04
Sex							
Male				*reference*			
Female	0.16	0.05	0.05	0.12	0.16	0.20	0.27
Day of week							
1				*reference*			
2	-0.08	0.11	-0.30	-0.15	-0.08	0.00	0.15
3	-0.05	0.11	-0.27	-0.13	-0.05	0.03	0.17
4	-0.26	0.12	-0.49	-0.34	-0.25	-0.18	-0.03
5	-0.26	0.11	-0.48	-0.34	-0.26	-0.18	-0.04
6	-0.28	0.13	-0.53	-0.37	-0.28	-0.20	-0.04
7	-0.29	0.13	-0.53	-0.37	-0.29	-0.20	-0.04
Employment							
Any work				*reference*			
No work	0.58	0.08	0.44	0.53	0.58	0.63	0.73
Household							
Single person				*reference*			
household with children	-0.41	0.12	-0.63	-0.49	-0.41	-0.33	-0.18
household without children	-0.31	0.10	-0.51	-0.38	-0.31	-0.25	-0.12
Vehicle ownership							
No vehicle				*reference*			
One or more vehicles	-2.66	0.19	-3.04	-2.78	-2.66	-2.53	-2.31
Weekly household income, equivalised							
0 – 500				*reference*			
501 – 817	0.03	0.11	-0.18	-0.05	0.02	0.10	0.24
817 – 1,157	0.16	0.11	-0.05	0.08	0.16	0.23	0.37
1,158 – 1,667	0.34	0.11	0.13	0.27	0.34	0.41	0.55
1,668 – 5,263	0.44	0.11	0.23	0.36	0.44	0.51	0.65
Random effects							
sd(u_household)	2.10	0.21	1.69	1.96	2.10	2.24	2.53
sd(u_meshblock)	0.41	0.07	0.29	0.36	0.41	0.46	0.55

Additional Table 2. Posterior estimates including covariates and random effects for hierarchical model predicting the log odds of driving for transport. Estimates are on the log-odds scale to facilitate comparing the magnitude of effect between measured covariates and random effects. The exponentiated log odds estimates are presented as adjusted odds ratios in Additional Table 6.

**Covariates**	**mean**	**sd**	**Posterior distribution percentiles**				
			**2.5%**	**25%**	**50%**	**75%**	**97.5%**
**ULI**	-0.16	0.01	-0.19	-0.17	-0.16	-0.15	-0.14
Age group							
18 – 29 year old				*reference*			
30 – 49 year old	0.48	0.12	0.25	0.40	0.48	0.56	0.70
50 – 64 year old	0.91	0.12	0.67	0.83	0.91	0.99	1.15
65 or older	0.76	0.15	0.47	0.66	0.76	0.86	1.05
Sex							
Male				*reference*			
Female	0.30	0.08	0.15	0.25	0.30	0.35	0.45
Day of week							
1				*reference*			
2	0.07	0.15	-0.23	-0.03	0.07	0.18	0.37
3	-0.01	0.15	-0.31	-0.11	-0.01	0.10	0.29
4	0.18	0.16	-0.13	0.07	0.18	0.28	0.47
5	0.19	0.16	-0.11	0.09	0.19	0.30	0.50
6	0.68	0.17	0.34	0.56	0.68	0.79	1.03
7	0.23	0.17	-0.09	0.12	0.23	0.35	0.57
Employment							
Any work				*reference*			
No work	-0.81	0.10	-1.01	-0.88	-0.81	-0.74	-0.62
Household							
Single person				*reference*			
household with children	0.06	0.16	-0.25	-0.05	0.06	0.17	0.38
household without children	-0.16	0.14	-0.43	-0.25	-0.16	-0.06	0.11
Vehicle ownership							
No vehicle				*reference*			
One or more vehicles	3.64	0.22	3.21	3.49	3.64	3.78	4.10
Weekly household income, equivalised							
0 – 500				*reference*			
501 – 817	0.16	0.14	-0.11	0.06	0.16	0.25	0.43
817 – 1,157	0.15	0.14	-0.13	0.05	0.15	0.25	0.43
1,158 – 1,667	0.21	0.14	-0.08	0.11	0.21	0.30	0.49
1,668 – 5,263	0.11	0.15	-0.18	0.01	0.11	0.21	0.40
Random effects							
sd(u_household)	2.77	0.35	2.15	2.53	2.76	3.00	3.49
sd(u_meshblock)	0.84	0.14	0.60	0.75	0.84	0.93	1.13

Additional Table 3. Posterior estimates including covariates and random effects for hierarchical model predicting the log odds of cycling for transport. Estimates are on the log-odds scale to facilitate comparing the magnitude of effect between measured covariates and random effects. The exponentiated log odds estimates are presented as adjusted odds ratios in Additional Table 7.

**Covariates**	**mean**	**sd**	**Posterior distribution percentiles**				
			**2.5%**	**25%**	**50%**	**75%**	**97.5%**
**ULI**	0.14	0.02	0.10	0.13	0.14	0.15	0.19
Age group							
18 – 29 year old				*reference*			
30 – 49 year old	0.35	0.23	-0.10	0.19	0.35	0.51	0.82
50 – 64 year old	0.22	0.24	-0.24	0.06	0.22	0.38	0.70
65 or older	-0.54	0.36	-1.25	-0.77	-0.54	-0.30	0.15
Sex							
Male				*reference*			
Female	-1.39	0.17	-1.73	-1.50	-1.39	-1.28	-1.08
Day of week							
1				*reference*			
2	-0.19	0.30	-0.79	-0.38	-0.19	0.02	0.39
3	-0.17	0.30	-0.76	-0.37	-0.18	0.03	0.41
4	0.05	0.29	-0.50	-0.15	0.05	0.24	0.62
5	-0.52	0.30	-1.12	-0.73	-0.52	-0.31	0.07
6	-0.87	0.35	-1.57	-1.10	-0.86	-0.63	-0.18
7	-0.38	0.34	-1.06	-0.60	-0.37	-0.15	0.28
Employment							
Any work				*reference*			
No work	-0.38	0.23	-0.84	-0.53	-0.38	-0.22	0.07
Household							
Single person				*reference*			
household with children	0.25	0.32	-0.38	0.03	0.25	0.46	0.90
household without children	0.51	0.29	-0.04	0.31	0.50	0.70	1.07
Vehicle ownership							
No vehicle				*reference*			
One or more vehicles	-1.05	0.39	-1.81	-1.32	-1.05	-0.79	-0.28
Weekly household income, equivalised							
0 – 500				*reference*			
501 – 817	0.17	0.33	-0.46	-0.04	0.17	0.39	0.83
817 – 1,157	0.53	0.32	-0.07	0.32	0.53	0.74	1.18
1,158 – 1,667	0.82	0.31	0.23	0.61	0.81	1.02	1.44
1,668 – 5,263	0.88	0.31	0.31	0.67	0.87	1.08	1.50
Random effects							
sd(u_household)	4.83	0.99	3.16	4.12	4.76	5.43	7.00
sd(u_meshblock)	1.81	0.44	1.03	1.50	1.77	2.08	2.76

Additional Table 4. Posterior estimates including covariates and random effects for hierarchical model predicting the log odds of public transport usage. Estimates are on the log-odds scale to facilitate comparing the magnitude of effect between measured covariates and random effects. The exponentiated log odds estimates are presented as adjusted odds ratios in Additional Table 8.

	mean	sd	Posterior distribution percentiles				
			2.5%	25%	50%	75%	97.5%
**ULI**	0.17	0.01	0.14	0.16	0.17	0.18	0.20
Age group							
18 – 29 year old				*reference*			
30 – 49 year old	-0.80	0.12	-1.05	-0.87	-0.79	-0.71	-0.55
50 – 64 year old	-1.37	0.13	-1.64	-1.46	-1.37	-1.28	-1.12
65 or older	-1.51	0.18	-1.86	-1.63	-1.51	-1.38	-1.17
Sex							
Male				*reference*			
Female	-0.19	0.08	-0.35	-0.24	-0.19	-0.13	-0.03
Day of week							
1				*reference*			
2	0.36	0.16	0.04	0.25	0.37	0.48	0.68
3	0.35	0.16	0.04	0.24	0.35	0.46	0.67
4	0.19	0.17	-0.14	0.08	0.19	0.30	0.51
5	0.12	0.17	-0.21	0.01	0.12	0.23	0.45
6	-0.87	0.20	-1.27	-1.00	-0.87	-0.73	-0.48
7	-1.21	0.22	-1.65	-1.36	-1.21	-1.06	-0.79
Employment							
Any work				*reference*			
No work	-0.05	0.11	-0.27	-0.12	-0.05	0.03	0.18
Household							
Single person				*reference*			
household with children	-0.32	0.17	-0.65	-0.43	-0.32	-0.21	0.01
household without children	0.07	0.14	-0.21	-0.03	0.07	0.17	0.36
Vehicle ownership							
No vehicle				*reference*			
One or more vehicles	-3.01	0.21	-3.43	-3.15	-3.01	-2.87	-2.61
Weekly household income, equivalised							
0 – 500				*reference*			
501 – 817	-0.13	0.16	-0.45	-0.24	-0.13	-0.02	0.19
817 – 1,157	0.09	0.16	-0.22	-0.02	0.09	0.19	0.40
1,158 – 1,667	0.16	0.16	-0.15	0.05	0.16	0.27	0.46
1,668 – 5,263	0.25	0.16	-0.05	0.14	0.25	0.36	0.56
Random effects							
sd(u_household)	2.20	0.33	1.62	1.97	2.19	2.41	2.91
sd(u_meshblock)	1.01	0.16	0.72	0.90	1.00	1.11	1.35

Additional Table 5. Adjusted odds ratios (AOR) including covariates for the hierarchical model predicting walking for transport. These are the exponentiated posterior estimates, corresponding to the log odds presented in Additional Table 1.

	AOR	Posterior distribution percentiles				
		**2.5%**	**25%**	**50%**	**75%**	**97.5%**
**ULI**	1.13	1.12	1.13	1.13	1.14	1.15
Age group						
18 – 29 year old			*reference*			
30 – 49 year old	0.86	0.72	0.81	0.86	0.91	1.03
50 – 64 year old	0.76	0.64	0.72	0.76	0.81	0.91
65 or older	0.77	0.62	0.71	0.77	0.83	0.97
Sex						
Male			*reference*			
Female	1.17	1.05	1.13	1.17	1.22	1.30
Day of week						
1			*reference*			
2	0.93	0.74	0.86	0.93	1.00	1.16
3	0.95	0.76	0.88	0.95	1.03	1.19
4	0.77	0.61	0.71	0.78	0.84	0.97
5	0.77	0.62	0.72	0.77	0.83	0.96
6	0.76	0.59	0.69	0.76	0.82	0.96
7	0.75	0.59	0.69	0.75	0.82	0.96
Employment						
Any work			*reference*			
No work	1.79	1.55	1.70	1.79	1.89	2.08
Household						
Single person			*reference*			
household with children	0.67	0.53	0.61	0.67	0.72	0.83
household without children	0.73	0.60	0.69	0.73	0.78	0.88
Vehicle ownership						
No vehicle			*reference*			
One or more vehicles	0.07	0.05	0.06	0.07	0.08	0.10
Weekly household income, equivalised						
0 – 500			*reference*			
501 – 817	1.03	0.84	0.96	1.02	1.10	1.27
817 – 1,157	1.17	0.95	1.09	1.17	1.26	1.44
1,158 – 1,667	1.40	1.14	1.31	1.40	1.51	1.74
1,668 – 5,263	1.54	1.25	1.43	1.55	1.66	1.92

Table 6. Adjusted odds ratios (AOR) including covariates for the hierarchical model predicting driving for transport. These are the exponentiated posterior estimates, corresponding to the log odds presented in Additional Table 2.

	AOR	Posterior distribution percentiles				
		**2.5%**	**25%**	**50%**	**75%**	**97.5%**
**ULI**	0.85	0.83	0.84	0.85	0.86	0.87
Age group						
18 – 29 year old						
30 – 49 year old	1.61	1.28	1.49	1.61	1.75	2.02
50 – 64 year old	2.49	1.95	2.29	2.48	2.70	3.16
65 or older	2.13	1.60	1.93	2.13	2.36	2.85
Sex						
Male						
Female	1.35	1.16	1.28	1.35	1.42	1.56
Day of week						
1						
2	1.07	0.80	0.97	1.07	1.19	1.45
3	0.99	0.73	0.90	1.00	1.11	1.34
4	1.19	0.87	1.07	1.19	1.33	1.61
5	1.21	0.89	1.09	1.21	1.35	1.64
6	1.97	1.41	1.75	1.97	2.21	2.80
7	1.26	0.91	1.13	1.26	1.42	1.77
Employment						
Any work						
No work	0.44	0.36	0.42	0.44	0.48	0.54
Household						
Single person						
household with children	1.07	0.78	0.95	1.06	1.19	1.46
household without children	0.86	0.65	0.78	0.86	0.94	1.11
Vehicle ownership						
No vehicle						
One or more vehicles	38.09	24.83	32.88	37.94	43.99	60.22
Weekly household income, equivalised						
0 – 500						
501 – 817	1.17	0.90	1.06	1.17	1.29	1.53
817 – 1,157	1.16	0.88	1.06	1.16	1.28	1.54
1,158 – 1,667	1.23	0.92	1.12	1.23	1.36	1.63
1,668 – 5,263	1.11	0.83	1.01	1.12	1.23	1.49

Additional Table 7. Adjusted odds ratios (AOR) including covariates for the hierarchical model predicting cycling for transport. These are the exponentiated posterior estimates, corresponding to the log odds presented in Additional Table 3.

	**AOR**	**Posterior distribution percentiles**				
		**2.5%**	**25%**	**50%**	**75%**	**97.5%**
**ULI**	1.15	1.11	1.13	1.15	1.17	1.20
Age group						
18 – 29 year old			*reference*			
30 – 49 year old	1.42	0.91	1.21	1.42	1.67	2.27
50 – 64 year old	1.25	0.79	1.06	1.25	1.46	2.00
65 or older	0.58	0.29	0.46	0.58	0.74	1.16
Sex						
Male			*reference*			
Female	0.25	0.18	0.22	0.25	0.28	0.34
Day of week						
1			*reference*			
2	0.83	0.45	0.68	0.83	1.02	1.48
3	0.84	0.47	0.69	0.84	1.03	1.50
4	1.05	0.61	0.86	1.05	1.27	1.85
5	0.59	0.33	0.48	0.59	0.73	1.07
6	0.42	0.21	0.33	0.42	0.53	0.83
7	0.69	0.35	0.55	0.69	0.86	1.32
Employment						
Any work			*reference*			
No work	0.69	0.43	0.59	0.69	0.81	1.07
Household						
Single person			*reference*			
household with children	1.28	0.68	1.03	1.28	1.59	2.45
household without children	1.66	0.96	1.36	1.65	2.02	2.92
Vehicle ownership						
No vehicle			*reference*			
One or more vehicles	0.35	0.16	0.27	0.35	0.45	0.75
Weekly household income, equivalised						
0 – 500			*reference*			
501 – 817	1.19	0.63	0.96	1.18	1.48	2.29
817 – 1,157	1.71	0.93	1.37	1.70	2.10	3.25
1,158 – 1,667	2.26	1.26	1.83	2.25	2.76	4.22
1,668 – 5,263	2.40	1.36	1.95	2.38	2.94	4.50

Additional Table 8. Adjusted odds ratios (AOR) including covariates for the hierarchical model predicting public transport usage. These are the exponentiated posterior estimates, corresponding to the log odds presented in Additional Table 4.

	**AOR**	**Posterior distribution percentiles**				
		**2.5%**	**25%**	**50%**	**75%**	**97.5%**
**ULI**	1.18	1.15	1.17	1.18	1.19	1.22
Age group						
18 – 29 year old			*reference*			
30 – 49 year old	0.45	0.35	0.42	0.45	0.49	0.57
50 – 64 year old	0.25	0.19	0.23	0.25	0.28	0.33
65 or older	0.22	0.16	0.20	0.22	0.25	0.31
Sex						
Male			*reference*			
Female	0.83	0.70	0.78	0.83	0.88	0.97
Day of week						
1			*reference*			
2	1.44	1.04	1.28	1.44	1.61	1.97
3	1.42	1.04	1.28	1.42	1.58	1.95
4	1.21	0.87	1.08	1.21	1.35	1.67
5	1.13	0.81	1.01	1.13	1.26	1.56
6	0.42	0.28	0.37	0.42	0.48	0.62
7	0.30	0.19	0.26	0.30	0.35	0.45
Employment						
Any work			*reference*			
No work	0.96	0.76	0.89	0.96	1.03	1.20
Household						
Single person			*reference*			
household with children	0.73	0.52	0.65	0.72	0.81	1.01
household without children	1.07	0.81	0.97	1.07	1.18	1.43
Vehicle ownership						
No vehicle			*reference*			
One or more vehicles	0.05	0.03	0.04	0.05	0.06	0.07
Weekly household income, equivalised						
0 – 500			*reference*			
501 – 817	0.88	0.64	0.79	0.88	0.98	1.21
817 – 1,157	1.09	0.80	0.98	1.09	1.21	1.48
1,158 – 1,667	1.17	0.87	1.05	1.17	1.31	1.58
1,668 – 5,263	1.29	0.95	1.15	1.29	1.43	1.75
